# The Alpha variant was not associated with excess nosocomial SARS-CoV-2 infection in a multi-centre UK hospital study

**DOI:** 10.1016/j.jinf.2021.09.022

**Published:** 2021-12

**Authors:** Florencia A.T. Boshier, Cristina Venturini, Oliver Stirrup, José Afonso Guerra-Assunção, Adela Alcolea-Medina, Angela H. Becket, Matthew Byott, Themoula Charalampous, Ana da Silva Filipe, Dan Frampton, Sharon Glaysher, Tabassum Khan, Raghavendran Kulasegara-Shylini, Beatrix Kele, Irene M. Monahan, Guy Mollett, Matthew Parker, Emanuela Pelosi, Paul Randell, Sunando Roy, Joshua F. Taylor, Sophie J. Weller, Eleri Wilson-Davies, Phillip Wade, Rachel Williams, Andrew J. Copas, Teresa Cutino-Moguel, Nick Freemantle, Andrew C. Hayward, Alison Holmes, Joseph Hughes, Tabitha W. Mahungu, Gaia Nebbia, Eleni Nastouli, David G. Partridge, Cassie F. Pope, James R. Price, Samuel C. Robson, Kordo Saeed, Gee Yen Shin, Thushan I. de Silva, Luke B. Snell, Emma C. Thomson, Adam A. Witney, Judith Breuer, COG-UK HOCI Variant Substudy consortium*, The COVID-19 Genomics UK (COG-UK) consortium

**Affiliations:** a Department of Infection, Immunity and Inflammation, UCL Great Ormond Street Institute of Child Health, University College London, London, United Kingdom; b Institute for Global Health, University College London, London, United Kingdom; c Department of Genetics and Genomic Medicine, UCL Great Ormond Street Institute of Child Health, University College London, London, United Kingdom; d Centre for Clinical Infection and Diagnostics Research, School of Immunology and Microbial Sciences, King's College London, London, United Kingdom; e Infection Sciences, Viapath, London, United Kingdom; f Centre for Enzyme Innovation, University of Portsmouth, Portsmouth PO1 2DT, United Kingdom; g School of Biological Sciences, University of Portsmouth, Portsmouth PO1 2DY, United Kingdom; h Advanced Pathogen Diagnostics Unit, University College London Hospitals NHS Foundation Trust, London, United Kingdom; i The Francis Crick Institute, London, United Kingdom; j MRC-University of Glasgow Centre for Virus Research, Glasgow, United Kingdom; k Division of Infection and Immunity, University College London, London, United Kingdom; l Portsmouth Hospitals University NHS Trust, Queen Alexandra Hospital, Portsmouth PO6 3LY, United Kingdom; m Division of Infection, The Royal London Hospital, Barts Health, United Kingdom; n Institute for Infection and Immunity, St George's University of London, Cranmer Terrace, London SW17 0RE, United Kingdom; o Sheffield Bioinformatics Core, The University of Sheffield, Sheffield, United Kingdom; p Sheffield Institute for Translational Neuroscience, The University of Sheffield, Sheffield, United Kingdom; q Sheffield Biomedical Research Centre, The University of Sheffield, Sheffield, United Kingdom; r Southampton Specialist Virology Centre, University Hospital Southampton NHS Foundation Trust, Southampton, United Kingdom; s Department of Infection and Immunity, North West London Pathology, London, United Kingdom; t Department of Microbiology, South West London Pathology, Jenner Wing, St. George's Hospital, Blackshaw Road, London SW17 0QT, United Kingdom; u Department of Virology, Royal Free London NHS Foundation Trust, London, United Kingdom; v Sheffield Teaching Hospitals NHS Foundation Trust, Sheffield, United Kingdom; w The Florey Institute for Host-Pathogen Interactions and Department of Infection, Immunity and Cardiovascular Disease, Medical School, University of Sheffield, Sheffield, United Kingdom; x https://www.cogconsortium.uk, United Kingdom; y Institute for Clinical Trials and Methodology, University College London, London, United Kingdom; z Institute of Epidemiology and Health Care, University College London, London, United Kingdom; aa Department of Infectious Disease, Faculty of Medicine, Imperial College London, United Kingdom; ab Imperial College Healthcare NHS Trust, Hammersmith Hospital, London, United Kingdom; ac Department of Infectious Diseases, Guy's and St Thomas’ Hospital NHS Foundation Trust, London, United Kingdom; ad Great Ormond Street Institute of Child Health, University College London, London, United Kingdom; ae Advanced Pathogen Diagnostics Unit, University College London Hospitals NHS Foundation Trust, London, United Kingdom; af Department of Clinical Virology, University College London Hospitals NHS Foundation Trust, London, United Kingdom; ag Infection Care Group, St George's University Hospitals NHS Foundation Trust, Blackshaw Road, London SW17 0QT, United Kingdom; ah Imperial College Healthcare NHS Trust, London, United Kingdom; ai School of Pharmacy and Biomedical Sciences, University of Portsmouth, Portsmouth PO1 2DT, United Kingdom; aj Department of Infection, University Hospital Southampton NHS Foundation Trust, Tremona Road, Southampton, United Kingdom; ak Faculty of Medicine, Clinical and Experimental Sciences, University of Southampton, Tremona Road, Southampton, United Kingdom; al Department of Microbiology, Great Ormond Street Hospital for Children NHS Foundation Trust, London, United Kingdom

**Keywords:** COVID-19, Transmissibility, Nosocomial outbreaks, Lineage B.1.1.7, Alpha variant, SARS-CoV-2, Variants of concern

## Abstract

**Objectives:**

Recently emerging SARS-CoV-2 variants have been associated with an increased rate of transmission within the community. We sought to determine whether this also resulted in increased transmission within hospitals.

**Methods:**

We collected viral sequences and epidemiological data of patients with community and healthcare associated SARS-CoV-2 infections, sampled from 16th November 2020 to 10th January 2021, from nine hospitals participating in the COG-UK HOCI study. Outbreaks were identified using ward information, lineage and pairwise genetic differences between viral sequences.

**Results:**

Mixed effects logistic regression analysis of 4184 sequences showed healthcare-acquired infections were no more likely to be identified as the Alpha variant than community acquired infections. Nosocomial outbreaks were investigated based on overlapping ward stay and SARS-CoV-2 genome sequence similarity. There was no significant difference in the number of patients involved in outbreaks caused by the Alpha variant compared to outbreaks caused by other lineages.

**Conclusions:**

We find no evidence to support it causing more nosocomial transmission than previous lineages. This suggests that the stringent infection prevention measures already in place in UK hospitals contained the spread of the Alpha variant as effectively as other less transmissible lineages, providing reassurance of their efficacy against emerging variants of concern.

## Introduction

At least four severe acute respiratory syndrome coronavirus 2 (SARS-CoV-2) lineages which resulted in strain replacement have been documented in the UK. For two of these, the Alpha variant (lineage B.1.1.7), and the Delta variant (lineage B.1.617.2), increased spread has been associated with increased variant transmissibility. The Alpha variant, which originated in the UK, was estimated to be up to 70% more transmissible than previously B.1 circulating variants and by March 2021 accounted for over 86% of cases in the UK.^1^, ^2^, ^3^, ^4^ The more recently emerged Delta variant is thought to be 40-60% more transmissible than the Alpha variant, and as of June 2021 replaced the latter as the most dominant variant in the UK.^5^^,^^6^ Both variants possess distinct mutations associated with increased transmissibility and antibody escape which might help explain their rise.^3^^,^^7^, ^8^, ^9^, ^10^

All SARS CoV-2 variants are associated with nosocomial transmission. For example, during the March-April 2020 peak of the COVID-19 outbreak it was estimated that up to 15% of inpatient cases were acquired in a healthcare setting.^11^, ^12^, ^13^, ^14^ With the recognition of highly transmissible variants, consideration has been given as to whether more stringent control measures would be needed to prevent increased spread in healthcare settings.^15^^,^^16^

This study aimed to determine if the reported increased community transmissibility of the Alpha variant is replicated in hospitals. To address this, we identified nosocomial outbreaks using data from the COVID-19 Genomics UK Consortium (COG-UK) Hospital Onset COVID-19 Infection (HOCI) study, which collected epidemiological information and viral sequences from healthcare/hospital acquired COVID-19 infections during the winter of 2020-21.

## Methods

### Sequence and patient meta-data

Data were collected as part of the COG-UK HOCI variant substudy from nine NHS hospitals across the UK, six of which were within London. The first SARS-CoV-2 positive sample from all inpatients, outpatient, A&E patients and healthcare workers (HCW), tested by hospital laboratories between 16th November 2020 and 10th January 2021, were sequenced. In addition metadata were collected on patient age, sex (f/m/other/unknown), date of hospital admission and ward location. Ethical approval for the HOCI study was provided by REC 20/EE/0118. Additional clinical details and comorbidities for this dataset are available elsewhere.^17^

Inpatients were classified into 3 groups: (i) patients admitted with SARS-CoV-2 (community-acquired infections, CAIs), (ii) those without symptoms of COVID 19 on admission, testing negative upon admission but testing positive between 3-7 days following admission (indeterminate healthcare-associated infections, HCAIs) and (iii) those without symptoms of COVID-19 on admission with a positive test >=8 days post-admission (probable/definite HCAIs).^18^ Sequence data were also available for patients who presented to hospital but were not admitted, hospital outpatients and healthcare workers. The non-inpatients groups are included in the evaluation of Alpha variant prevalence only.

### SARS-CoV-2 sequencing

Samples were sequenced by Oxford Nanopore Technologies (ONT)-based or Illumina-based methods as part of the COG-UK consortium.^19^ To maximise success 3 of 9 labs sequenced only those samples with qPCR cycle thresholds (Ct) values of ≤32 or equivalent, corresponding to 54% of samples (2268/4184). Sequences were assigned to lineages using COG-UK Pangolin (date 2021-04-14).^20^ The GISAID and/or ENA accession number of 3589 sequences which are publicly available are in supplementary Table 1.

### Prevalence in community testing (Pillar 2) from COG-UK

The number of samples in the COG-UK dataset collected between 16th November 2020 and 10th January 2021 from community areas, local to participating hospitals (i.e. shared adm2 designation), was tallied by week.^21^

### Statistical analysis

Differences between patient groups in the prevalence of the Alpha variant among positive samples were evaluated using mixed effects logistic regression.^22^ CAI or HCAI, sex, age and sample week were included as predictive variables. Parameters for sample weeks were fitted separately for London sites compared with other sites grouped, and random intercept terms were included for each hospital and for weekly periods nested within hospitals. This analysis was also repeated including only the London sites.

Outbreak analyses were conducted using sequences with greater than 90% coverage across the SARS-CoV-2 genome (1043 sequences). Sequence diversity was measured by pairwise distance, defined as the number of single nucleotide polymorphisms (SNPs) differences between two sequences (excluding Ns), calculated in the R `ape` package.^23^ The summary results were then grouped by lineage. To determine whether sequences were part of a nosocomial outbreak, we only focused on probable/definite HCAIs diagnosed ≥8 days post-admission. Cases occurring on the same wards (excluding known COVID-19 wards), with a pairwise distance of 0 (i.e. identical sequences) and within a time window of ⩽ 7 days were considered linked and part of the same outbreak. We also included, as independent outbreaks, all samples not linked to any other (i.e. one unlinked sample irrespective of time and location will count as an outbreak of size 1). As these patients all acquired the infection in hospital, they are likely to represent nosocomial transmission (for example from other patients or HCWs whose virus was not sequenced or did not achieve adequate coverage).

All analyses were conducted in R version 4.0.2, using tidyverse collection of packages and other statistical packages such as lme4,^22^ jtools^24^ and rcompanion.^25^ All plots were generated using ggplot2.^26^

## Results

### Study dataset

Between November 16th 2020 and January 10th 2021 SARS-CoV-2 RNA positive upper respiratory tract samples from 4184 subjects were successfully sequenced, including 2455 inpatients, 450 outpatients, 1166 HCWs and 113 (4.4 %) with unknown status. Of the inpatients, 1666 (64.9 %) were hospitalised with community-acquired infection, 215 (8.4 %) with indeterminate HCAI and 574 (22.4 %) with probable/definite HCAI, (Table 1). In total, 2058 samples were the Alpha variant, 4 samples were the Beta variant (lineage B.1.351) and 2122 were of lineages not designated variants of concern.The two most prominent lineages across the dataset were B.1.1.7 (the Alpha variant) and B.1.177. This was also true when restricting to HCAI samples alone (Supplementary Fig. 1).

**Table 1:** Proportion of SARS-CoV-2 due to the Alpha variant for all sequenced samples.

	Alpha variant (n=2058)	Non-Alpha variant (n=2126)	Total (n=4184)
**Age [mean (sd)]**			
	53.4 (21.8)	58 (22.6)	55.7 (22.3)
Missing	0	1	1
**Sex**			
Female	1109 (48.6)	1175 (51.4)	2,284 (100.0)
Male	938 (49.8)	944 (50.2)	1,882 (100.0)
Missing	11	7	18
**Week starting:**			
16/11/2020	22 (8.5)	238 (91.5)	260 (100.0)
23/11/2020	50 (15.0)	284 (85.0)	334 (100.0)
30/11/2020	83 (20.4)	324 (79.6)	407 (100.0)
07/12/2020	128 (30.0)	299 (70.0)	427 (100.0)
14/12/2020	312 (45.7)	370 (54.3)	682 (100.0)
21/12/2020	411 (57.2)	307 (42.8)	718 (100.0)
28/12/2020	648 (75.2)	214 (24.8)	862 (100.0)
04/01/2021	404 (81.8)	90 (18.2)	494 (100.0)
**Patient Class**			
Outpatients	250 (55.6)	200 (44.4)	450 (100.0)
Any HCW	559 (47.9)	607 (52.1)	1,166 (100.0)
Inpatients	1182 (48.1)	1273 (51.9)	2,455 (100.0)
CAI*	926 (55.6)	740 (44.4)	1,666 (100.0)
Indeterminate HCAI†	56 (26.0)	159 (74.0)	215 (100.0)
Probable/definite HCAI‡	200 (34.8)	374 (65.2)	574 (100.0)
Unknown category	67 (59.3)	46 (40.7)	113 (100.0)
**Region**			
Glasgow	91 (31.6)	197 (68.4)	288 (100.0)
Hampshire	288 (66.2)	147 (33.8)	435 (100.0)
London	1480 (65.6)	775 (34.4)	2,255 (100.0)
South Yorkshire	199 (16.5)	1007 (83.5)	1,206 (100.0)

⁎ Diagnosed at or ≤2 days from admission.

† Diagnosed 3-7 days from admission.

‡ Diagnosed ≥8 days from admission. CAI, community-acquired infection; HCAI, healthcare-associated infection; HCW, healthcare worker.

Data from laboratories not using Ct or equivalent thresholds confirmed that the proportions of the Alpha variant and non-Alpha variant viruses did not differ in samples with Ct values <=32 (Supplementary Fig. 2, Chi-square test p=0.16).

### Prevalence of the Alpha variant

**Fig. 1:**
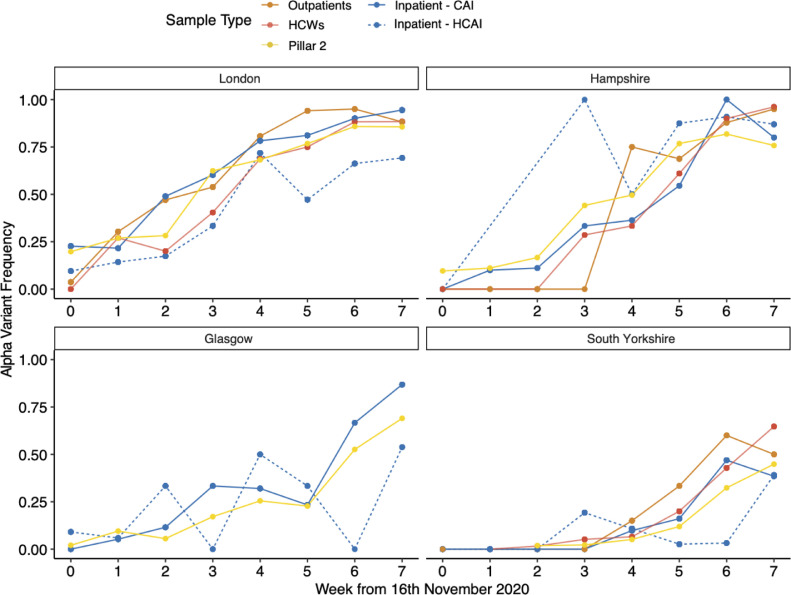
Prevalence over time of the Alpha variant in hospitalized patients, healthcare workers (HCWs) and community samples (Pillar 2 data as described in methods) from different geographical regions in the UK. Hospitalized patients are displayed according to community-acquired infection (CAI) (diagnosed at or ≤2 days from admission) or healthcare-associated infection (HCAI) (diagnosed ≥3 days from admission).

The prevalence of the Alpha variant was highest in London and Hampshire (South of England), but substantially increased at all sites over the study period (Fig. 1). On mixed effects logistic regression analysis of the Alpha variant, using 4165 samples with complete metadata, samples from HCWs (OR 0.78, 95 CI% 0.60 to 1.01), indeterminate HCAIs (OR 0.45, 95 CI% 0.30 to 0.70) or probable/definite HCAI (0.45, 0.34 to 0.59) were less likely to be identified as the Alpha variant compared to CAIs than non-Alpha variant. Suggesting that the proportion of hospital-acquired infections due to the Alpha variant was lower in any given week than the proportion among those presenting to hospital with community-acquired infection. However, changes in the frequency of the Alpha variant in CAIs correlated with those in HCAIs on a regional basis (Pearson's correlation coefficient in London 0.90, 95% CI: 0.54-0.98, p-value<0.01, outside London 0.88, 95% CI 0.45-0.98, p-value<0.05) (Supplementary Fig. 3a). This relationship was confirmed also between HCAIs and community data from the general population (Pillar2, Supplementary Fig. 3b). Following the rapid growth of the Alpha variant within the community and hospitals, we observed a decrease of other lineages. In particular, B.1.177, which was the dominant strain in Europe before November 2020,^27^^,^^28^ showed a correlation between CAIs and HCAIs (overall correlation 0.85) and an opposite trend to the Alpha variant with frequencies decreasing overtime (Supplementary Fig. 4).

### Pairwise distance in HCAI

To help define outbreaks within hospitals, we used the sequence diversity within outbreaks involving patients with defined probable/definite HCAIs. We first compared the genetic distance among the Alpha variant sequences and separately among non-Alpha variant sequences of the same lineage. We found the mean pairwise distance (measured as number of SNPs difference) was lower between the Alpha variant samples than between samples from other lineages (mean=6.75 SNPs (95% CI 6.74-6.78) vs mean=8.01 SNPs (95% CI 7.95–8.07), Mann-Whitney U test p <0.05, Supplementary Fig. 5). We next considered only viruses from patients who had very likely acquired their infection in hospital (i.e. probable/definite HCAIs). Excluding wards that were used for cohorting COVID-19 patients, the mean pairwise distance between sequences from patients on the same ward was higher for the Alpha variant acquired in hospital than for non-Alpha (mean=1.95 SNPs (95% CI 1.64–2.27) vs mean = 0.71 SNPS (95% CI 0.635-0.78), Mann-Whitney U test p <0.05). However, for both the Alpha variant and non-Alpha variants the pairwise distance between samples in the same ward was low.

### Outbreaks

Given the low diversity observed within wards, and in agreement with previous studies,^14^ a stringent definition was applied to define linked infections. Samples were considered linked, and part of the same outbreak, when the the sequences were completely identical and occurred on the same ward within a period of 7 days. Outbreaks of size one, corresponding to samples not linked to any other sample, were allowed. The 7 day threshold is consistent with evidence that most people become symptomatic 7 days after exposure.^29^^,^^30^ This choice was also inline with previous transmission studies.^16^ The impact of allowing for multi-ward outbreaks and varying the time period and the pairwise SNP differences defining an outbreak was tested in a sensitivity analysis.

**Fig. 2:**
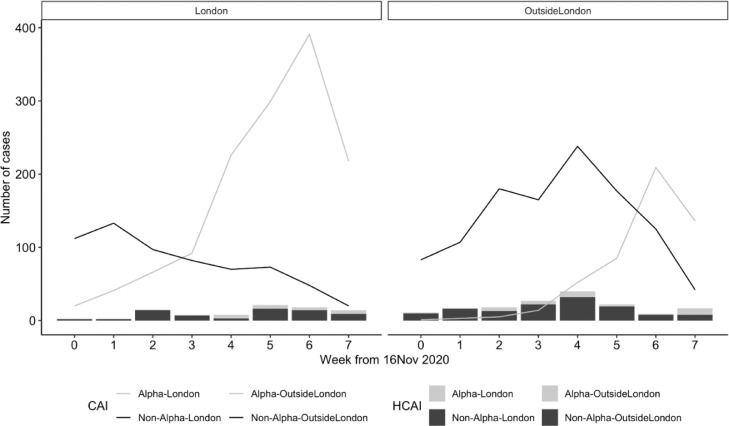
Barplot showing number of HOCI patients involved in outbreaks by week and location, coloured by variant (Alpha vs non-Alpha). Line-chart represents the number of CAI (community-acquired infections, including inpatients, outpatient, A&E patients and healthcare workers) overtime coloured by variant (Alpha variant presence/absence).

Ward data was available for a total of 497 probable/definite HCAI patients. A total of 83 outbreaks were identified (by the above definition) caused by any lineage across all hospitals, 19 of which were caused by the Alpha variant. Outbreaks caused by the Alpha variant in hospitals increased with time, associated with the changing prevalence of the Alpha variant within the community (Fig. 2). In contrast outbreaks due to other lineages decreased in line with reduced circulation of those lineages in the community. Whilst this trend is observed both within and outside London, the dominance of the Alpha variant outbreaks occurs earlier within London, reflecting the earlier rise in the community.

**Fig. 3:**
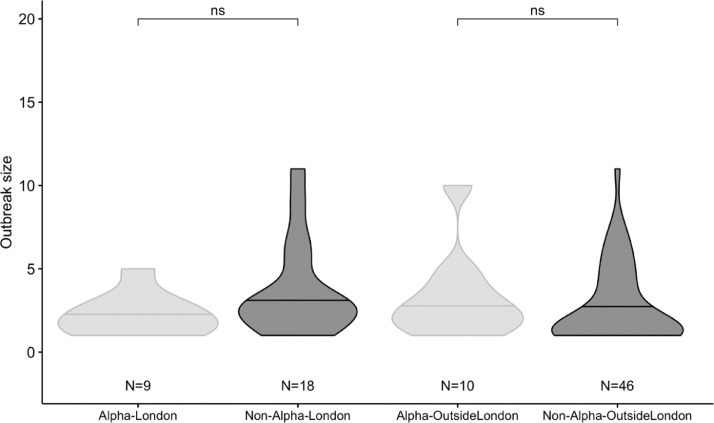
Violin plot showing the size of outbreaks in hospital-onset COVID-19 infection patients for four categories: outbreaks caused by the Alpha variant in London and other locations and outbreaks caused by other lineages in London and outside London. Colour represents lineages: in lighter grey the Alpha variant and in black non-Alpha variant. Non-parametric global Kruskal-Wallis p-value=0.27, pairwise comparisons (Mann-Whitney) non-significant. The number below each violin shows the number of clusters/outbreaks for that category.

The sizes of outbreak clusters within hospitals caused by the Alpha variant and by other lineages were compared. The total number of probable/definite HCAI patients in a single outbreak ranged from 1 to 11. There was no significant difference in the number of patients involved in outbreaks caused by the Alpha variant compared to outbreaks caused by other lineages (global Kruskal-Wallis p-value=0.27, pairwise comparisons non-significant, Fig. 3). The mean size for the Alpha variant outbreaks was 2.22 in London (95% CI 1.22–3.22) and 3.30 in other locations (95% CI 1.39–5.21). Outbreaks of non-B.1.1.7 lineages had a mean size of 3.72 and 2.78 in London and outside respectively (95% CI 2.32–5.13 in London and 95% CI 2.08–3.49 outside). These conclusions were unchanged, by the sensitivity analyses (Supplementary Fig. 6).

## Discussion

Nosocomial transmission continues to present a major challenge to the control of SARS-CoV-2 infection. Overall SARS-CoV-2 acquired in hospitals is estimated to have accounted for up to 20% COVID-19 inpatient cases during the first wave.^31^ Recent data from Scotland suggest that up to 36% of severe COVID-19 is associated with recent exposure in hospital (from 1 March 2020 to 28 January 2021).^32^ This is in line with the proportions identified in our data, with 22.4% of inpatients having probable/definite HCAI and 8.4% having indeterminate HCAI across all sites. The emergence of new variants with evidence of greater transmissibility in the community presents a potentially increased threat of nosocomial transmission leading to calls for better protection for staff and patients.^15^

Using detailed metadata on community and healthcare-acquired infections from 2455 inpatients in 9 hospitals across the UK linked to genomic data sequenced during the winter of 20/21 as part of COG-UK HOCI study, logistic regression analysis showed that having a healthcare-acquired infection was predictive of non-Alpha variants. This implies that the Alpha variant was not spreading faster within hospitals than in the community (Table 2). This finding was despite a rise in numbers of COVID-19 cases among both inpatients and the community, with an increasing proportion caused by the Alpha variant (Fig. 1). As has been previously reported, the total numbers of HCAIs were closely correlated with the rising numbers of cases in the community and the increase in HCAI infections caused by the Alpha variant also correlated with increasing prevalence of the Alpha variant overall.^29^

**Table 2:** Multivariable mixed effects logistic regression for prediction of being infected with the Alpha variant among positive samples sequenced by hospital labs.

	All samples		London sites only	
	OR (95% CI)	p-value	OR (95% CI)	p-value
**Age**		0.03		0.09
	0.99(0.99–1.00)		1.00(0.99–1.00)	
**Sex**		0.51		0.43
Female	*Reference*		*Reference*	
Male	0.95(0.80–1.12)		0.92(0.74–1.14)	
**Patient class**		<0.001		<0.001
Inpatient (CAI)†	*Reference*		*Reference*	
A&E attendee	1.35(0.87–2.09)		1.25 (0.76–2.05)	
Outpatient	0.86(0.58–1.26)		0.78 (0.47–1.32)	
Any HCW	0.78(0.60–1.01)		0.67 (0.48–0.93)	
Indeterminate HCAI‡	0.45(0.30–0.70)		0.33 (0.19–0.58)	
Probable/definite HCAI‡	0.45(0.34–0.59)		0.29 (0.20–0.41)	
Unknown	2.46(1.41–4.30)		3.19 (1.28–7.92)	
**Week starting:**	**Mean Prop. London#**	**Mean Prop. Elsewhere#**	**Mean Prop.#**	
16/11/2020	0.14(0.09–0.21)	0.02(0–0.07)	0.08(0.06–0.13)	
23/11/2020	0.23(0.17–0.30)	0.03(0.01–0.08)	0.15(0.11–0.19)	
30/11/2020	0.36(0.30–0.44)	0.05(0.03–0.09)	0.20(0.17–0.25)	
07/12/2020	0.50(0.43–0.57)	0.10(0.07–0.15)	0.30(0.26–0.35)	
14/12/2020	0.76(0.67–0.81)	0.18(0.14–0.22)	0.46(0.42–0.50)	
21/12/2020	0.77(0.72–0.81)	0.30(0.25–0.36)	0.57(0.53–0.60)	
28/12/2020	0.86(0.83–0.89)	0.60(0.55–0.65)	0.75(0.72–0.78)	
04/01/2021	0.88(0.84–0.92)	0.74(0.67–0.79)	0.82(0.78–0.84)	

† Diagnosed at or ≤2 days from admission.

‡ Diagnosed 3-7 days from admission. Diagnosed ≥8 days from admission. **#**Estimate of proportion infected with the Alpha variant from model for a 55-year-old male inpatient admitted with COVID-19. CAI, community-acquired infection; HCAI, healthcare-associated infection; HCW, healthcare worker; OR, odds ratio.

We made use of the genomic data and detailed information on hospital acquired infections to better identify and quantify linked hospital infections. The definition of an outbreak was considered carefully. Previous outbreak data suggest that the mutation rate of SARS-CoV-2 is low, with an average of less than one fixed mutation occurring for each transmission.^33^ Nonetheless, up to 2 single nucleotide differences have been described in viruses that are known to be part of a single nosocomial outbreak.^34^ In our data, we noted very little genetic diversity across the Alpha variant (Supplementary Fig. 5), reflecting the rapid expansion and selective sweep that occurred as the variant rapidly spread. We therefore chose a stringent definition of linked infections, requiring identical sequences and included only patients with a high likelihood of having acquired their infection in hospital (i.e. probable or definite hospital onset SARS-CoV-2 infection). We also restricted putatively linked cases to those on the same ward and within a time window of 7 days to further increase the specificity of outbreak definition. Within these constraints, the genomic data failed to identify a difference between the size of outbreaks occurring on wards between the Alpha variant and previously circulating lineages.

However, the outbreak definition implemented in our primary analysis is rather stringent. First, as we lack complete records of patients movement, we potentially exclude linked cases in different wards, for example patients who were infected by the same health-care worker or patients who moved before/after diagnosis. Second, our choice of a 7 days window is rather conservative, considering that estimates of the incubation period vary with some outbreak studies opting for a larger period of 14 days.^29^ Third, using only identical sequences we could bias against lineages with smaller diversity. To assess the impact of our parameters’ choice and the robustness of our results, we carried out a sensitivity analysis varying our parameters to link cases. Allowing for multi-ward outbreaks, increasing the numbers of SNP differences to two and varying the time interval for defining linked cases (0, 7 and 14 days) failed to change the findings.

There are a number of limitations to our work. First, we were not able to sequence all positive cases. Five of nine centres only sequenced samples with PCR cycle thresholds of 32 and below i.e. higher viral loads. Notably though, sequencing of 694 cases, from three labs not using Ct thresholds with available Ct data, did not find any difference in the distribution of genotypes in samples with Ct values below and above 32 (supplementary Fig. 1). A second limitation of our work is that towards the end of the study all three trusts outside London were using a sequence reporting tool (SRT), as part of the HOCI study,^33^ rather than phylogenetic analysis alone to help determine whether cases were part of linked outbreaks. It is not known whether the SRT may have limited the extent of outbreaks as data processing and analysis for the HOCI study is still ongoing. Finally, this study was not designed to account for use of personal protective equipment (PPE), aerosol generating procedures (AGP) or ventilation which may also impact transmission.

In summary notwithstanding its greater transmissibility in the community, we find no evidence to support the Alpha variant as having caused more nosocomial transmission than previous variants. This suggests that the stringent infection prevention measures already in place in UK hospitals are similarly effective at containing the spread of SARS-CoV-2 in a healthcare setting irrespective of its transmissibility. This finding implies that ongoing nosocomial spread of SARS-CoV-2 is likely to be influenced by factors such as fixed estate, e.g. building infrastructure, beds in bays, shared facilities and ventilation, which are not readily mitigated by the existing infection prevention and control (IPC) measures. However, there is some reassurance that currently implemented IPC measures are likely to be as effective against more transmissible variants.

## Funding

COG-UK HOCI funded by COG-UK consortium. The COG-UK consortium is supported by funding from the Medical Research Council (MRC) part of UK Research & Innovation (UKRI), the National Institute of Health Research (NIHR) and Genome Research Limited, operating as the Wellcome Sanger Institute.

## Appendix Supplementary materials

Image, application 1

Image, application 2

Image, application 3

## References

[1] Volz E., Mishra S., Chand M. (2020). Transmission of SARS-CoV-2 Lineage B.1.1.7 in England: Insights from linking epidemiological and genetic data. medRxiv.

[2] Davies N.G., Abbott S., Barnard R.C. (2021). Estimated transmissibility and impact of SARS-CoV-2 lineage B.1.1.7 in England. Science.

[3] O’Toole Á, Hill V, Pybus OG, Watts A, Bogoch II, Khan K, et al. **Tracking the international spread of SARS-CoV-2 lineages B.1.1.7 and B.1.351/501Y-V2 with grinch [Internet]. Wellcome Open Research; 2021 [cited 2021 Oct 21]. Available from:** **https://wellcomeopenresearch.org/articles/6-121** 10.12688/wellcomeopenres.16661.1 PMC8176267 34095513

[4] (2020). Lineage-specific growth of SARS-CoV-2 B.1.1.7 during the English national lockdown - SARS-CoV-2 coronavirus /nCoV-2019 Genomic Epidemiology. Virological.

[5] Public Health England. Confirmed cases of COVID-19 variants identified in UK. GOV.UK. Available at: https://www.gov.uk/government/news/confirmed-cases-of-covid-19-variants-identified-in-uk (accessed June 29, 2021).

[6] Scientific Advisory Group for Emergencies. SPI-M-O: Consensus statement on COVID-19, 3 June 2021. GOV.UK. Available at: https://www.gov.uk/government/publications/spi-m-o-consensus-statement-on-covid-19-3-june-2021 (accessed June 29, 2021).

[7] Kemp S.A., Collier D.A., Datir R.P. (2021). SARS-CoV-2 evolution during treatment of chronic infection. Nature.

[8] Wise J. (2020). Covid-19: New coronavirus variant is identified in UK. BMJ.

[9] Starr T.N., Greaney A.J., Dingens A.S., Bloom JD. (2021). Complete map of SARS-CoV-2 RBD mutations that escape the monoclonal antibody LY-CoV555 and its cocktail with LY-CoV016. Cell Rep Med.

[10] Zhang W., Davis B.D., Chen S.S., Sincuir Martinez J.M., Plummer J.T., Vail E. (2021). Emergence of a novel SARS-CoV-2 variant in Southern California. JAMA.

[11] Rickman H.M., Rampling T., Shaw K. (2021). Nosocomial transmission of Coronavirus Disease 2019: a retrospective study of 66 hospital-acquired cases in a London teaching hospital. Clin Infect Dis.

[12] Wang D., Hu B., Hu C. (2020). Clinical characteristics of 138 hospitalized patients with 2019 novel Coronavirus–infected pneumonia in Wuhan, China. JAMA.

[13] Bhattacharya A., Collin S.M., Stimson J., et al. Healthcare-associated COVID-19 in England: a national data linkage study. Infect Dis (except HIV/AIDS), 2021 DOI:10.1101/2021.02.16.21251625. 10.1016/j.jinf.2021.08.039 PMC8404398 34474055

[14] Meredith L.W., Hamilton W.L., Warne B. (2020). Rapid implementation of SARS-CoV-2 sequencing to investigate cases of health-care associated COVID-19: a prospective genomic surveillance study. Lancet Infect Dis.

[15] Oliver OD.D (2021). Could we do better on hospital acquired covid-19 in a future wave?. BMJ.

[16] Duverger C., Souyri V., Monteil C. (2021). Controlling healthcare-associated transmission of SARS-CoV-2 variant of concern 202012/01 in a large hospital network. J Hosp Infect.

[17] Stirrup O.T., Boshier F.A.T., Venturini C. (2021). SARS-CoV-2 lineage B.1.1.7 is associated with greater disease severity among hospitalised women but not men. BMJ Open Respir Res.

[18] UK Health Security Agency. COVID-19: epidemiological definitions of outbreaks and clusters. GOV.UK. Published the Aug 7, 2020. Available at: https://www.gov.uk/government/publications/covid-19-epidemiological-definitions-of-outbreaks-and-clusters (accessed Feb 9, 2021).

[19] COVID-19 Genomics UK Consortium. COVID-19 ARTIC v3 Illumina library construction and sequencing protocol (published Nov 4, 2020). Available at: https://www.protocols.io/view/covid-19-artic-v3-illumina-library-construction-an-bibtkann

[20] O’Toole Á, Scher E, Underwood A, Jackson B, Hill V, McCrone JT, et al. **Assignment of epidemiological lineages in an emerging pandemic using the pangolin tool. Virus Evolution [Internet]. 2021 Oct 1 [cited 2021 Oct 21];7(2). Available from:** 10.1093/ve/veab064 PMC8344591 34527285

[21] COG-UK Consortium. Public data & analysis (2021). Available at: https://www.cogconsortium.uk/tools-analysis/public-data-analysis-2/

[22] Bates D., Mächler M., Bolker B., Walker S. (2015). Fitting linear mixed-effects models using lme4. J Stat Softw.

[23] Paradis E., Schliep K. (2019). ape 5.0: an environment for modern phylogenetics and evolutionary analyses in R. Bioinformatics.

[24] Long JA, Jtools: Analysis and presentation of social scientific data. R package version 2.1.0. Available at: https://jtools.jacob-long.com/ (accessed June 16, 2021).

[25] Mangiafico, S.S. Summary and Analysis of Extension Program Evaluation in R, version 1.18.8., 2016 rcompanion.org/handbook/. (Pdf version: rcompanion.org/documents/RHandbookProgramEvaluation.pdf.)

[26] Wickham H., Averick M., Bryan J. (2019). Welcome to the Tidyverse. J Open Source Softw.

[27] Di Giallonardo F., Puglia I., Curini V. (2021). Emergence and spread of SARS-CoV-2 Lineages B.1.1.7 and P.1 in Italy. Viruses.

[28] Jackson B, Rambaut A, Pybus OG (2021). Recombinant SARS-CoV-2 genomes involving lineage B.1.1.7 in the UK - SARS-CoV-2 coronavirus /SARS-CoV-2 molecular evolution. Virological.

[29] Snell L.B., Fisher C.L., Taj U. (2020). Combined epidemiological and genomic analysis of nosocomial SARS-CoV-2 transmission identifies community social distancing as the dominant intervention reducing outbreaks. medRxiv.

[30] Wiersinga W.J., Rhodes A., Cheng A.C., Peacock S.J., Pathophysiology PHC. (2020). Transmission, diagnosis, and treatment of Coronavirus disease 2019 (COVID-19): a review. JAMA.

[31] Evans S, Agnew E, Vynnycky E (2021). The impact of testing and infection prevention and control strategies on within-hospital transmission dynamics of COVID-19 in English hospitals. Philos. Trans. R. Soc. B Biol. Sci..

[32] McKeigue PM, McAllister DA, Caldwell D (2021). **Relation of severe COVID-19 in Scotland to transmission-related factors and risk conditions eligible for shielding support: REACT-SCOT case-control study**. BMC Medicine.

[33] Stirrup O., Hughes J., Parker M. (2021). Rapid feedback on hospital onset SARS-CoV-2 infections combining epidemiological and sequencing data. eLife.

[34] Rockett R.J., Arnott A., Lam C. (2020). Revealing COVID-19 transmission in Australia by SARS-CoV-2 genome sequencing and agent-based modeling. Nat Med.

