# Supplementary material for: The Alpha variant was not associated with excess nosocomial SARS-CoV-2 infection in a multi-centre UK hospital study
Source: J Infect. 2021 Dec;83(6):693–700. doi: 10.1016/j.jinf.2021.09.022 (PMC8487101; doi:10.1016/j.jinf.2021.09.022)

**Abstract [250/250]**

Objectives

Recently emerging SARS-CoV-2 variants have been associated with an increased rate of transmission within the community. We sought to determine whether this also resulted in increased transmission within hospitals.

Methods

We collected viral sequences and epidemiological data of patients with community and healthcare associated SARS-CoV-2 infections, sampled from 16th November 2020 to 10th January 2021, from nine hospitals participating in the COG-UK HOCI study. Outbreaks were identified using ward information, lineage and pairwise genetic differences between viral sequences.

Results

Mixed effects logistic regression analysis of 4184 sequences showed healthcare-acquired infections were no more likely to be identified as the Alpha variant than community acquired infections. Nosocomial outbreaks were investigated based on overlapping ward stay and SARS-CoV-2 genome sequence similarity. There was no significant difference in the number of patients involved in outbreaks caused by the Alpha variant compared to outbreaks caused by other lineages.

Conclusions

We find no evidence to support it causing more nosocomial transmission than previous lineages. This suggests that the stringent infection prevention measures already in place in UK hospitals contained the spread of the Alpha variant as effectively as other less transmissible lineages, providing reassurance of their efficacy against emerging variants of concern.

**Funding**

COG-UK HOCI funded by COG-UK consortium. The COG-UK consortium is supported by funding from the Medical Research Council (MRC) part of UK Research & Innovation (UKRI), the National Institute of Health Research (NIHR) and Genome Research Limited, operating as the Wellcome Sanger Institute.

**Key words:** COVID-19; transmissibility ;nosocomial outbreaks; lineage B.1.1.7; Alpha variant; SARS-CoV-2; variants of concern

**40 word summary:** This UK multicentre study found no evidence to support the Alpha variant as having caused more nosocomial transmission that previous SARS-CoV-2 variants. This provides some reassurance that currently implemented IPC measures may be as effective against more transmissible variants.

**[2663/3000]**

**Introduction**

At least four severe acute respiratory syndrome coronavirus 2 (SARS-CoV-2) lineages which resulted in strain replacement have been documented in the UK. For two of these, the Alpha variant (lineage B.1.1.7), and the Delta variant (lineage B.1.617.2), increased spread has been associated with increased variant transmissibility. The Alpha variant, which originated in the UK, was estimated to be up to 70% more transmissible than previously B.1 circulating variants and by March 2021 accounted for over 86% of cases in the UK[^1–4^](https://www.zotero.org/google-docs/?ksirZX). The more recently emerged Delta variant is thought to be 40-60% more transmissible than the Alpha variant, and as of June 2021 replaced the latter as the most dominant variant in the UK[^5,6^](https://www.zotero.org/google-docs/?7BjvPc). Both variants possess distinct mutations associated with increased transmissibility and antibody escape which might help explain their rise[^3,7–10^](https://www.zotero.org/google-docs/?VDbml5).

All SARS CoV-2 variants are associated with nosocomial transmission. For example, during the March-April 2020 peak of the COVID-19 outbreak it was estimated that up to 15% of inpatient cases were acquired in a healthcare setting[^11–14^](https://www.zotero.org/google-docs/?cixz1j). With the recognition of highly transmissible variants, consideration has been given as to whether more stringent control measures would be needed to prevent increased spread in healthcare settings[^15,16^](https://www.zotero.org/google-docs/?Ux4Cqq).

This study aimed to determine if the reported increased community transmissibility of the Alpha variant is replicated in hospitals. To address this, we identified nosocomial outbreaks using data from the COVID-19 Genomics UK Consortium (COG-UK) Hospital Onset COVID-19 Infection (HOCI) study, which collected epidemiological information and viral sequences from healthcare/hospital acquired COVID-19 infections during the winter of 2020-21.

**Methods**

Sequence and patient meta-data

Data were collected as part of the COG-UK HOCI variant substudy from nine NHS hospitals across the UK, six of which were within London. The first SARS-CoV-2 positive sample from all inpatients, outpatient, A&E patients and healthcare workers (HCW), tested by hospital laboratories between 16^th^ November 2020 and 10^th^ January 2021, were sequenced. In addition metadata were collected on patient age, sex (f/m/other/unknown), date of hospital admission and ward location. Ethical approval for the HOCI study was provided by REC 20/EE/0118. Additional clinical details and comorbidities for this dataset are available elsewhere[^17^](https://www.zotero.org/google-docs/?pYcdUf).

Inpatients were classified into 3 groups: i) patients admitted with SARS-CoV-2 (community-acquired infections, CAIs), ii) those without symptoms of COVID 19 on admission, testing negative upon admission but testing positive between 3-7 days following admission (indeterminate healthcare-associated infections, HCAIs) and iii) those without symptoms of COVID-19 on admission with a positive test >=8 days post-admission (probable/definite HCAIs)[^18^](https://www.zotero.org/google-docs/?WVKc82). Sequence data were also available for patients who presented to hospital but were not admitted, hospital outpatients and healthcare workers. The non-inpatients groups are included in the evaluation of Alpha variant prevalence only.

SARS-CoV-2 sequencing

Samples were sequenced by Oxford Nanopore Technologies (ONT)-based or Illumina-based methods as part of the COG-UK consortium[^19^](https://www.zotero.org/google-docs/?jY13R4). To maximise success 3 of 9 labs sequenced only those samples with qPCR cycle thresholds (Ct) values of ≤32 or equivalent, corresponding to 54% of samples (2268/4184). Sequences were assigned to lineages using COG-UK Pangolin (date 2021-04-14)[^20^](https://www.zotero.org/google-docs/?nSn1Y1). The GISAID and/or ENA accession number of 3589 sequences which are publicly available are in supplementary table 1.

Prevalence in community testing (Pillar 2) from COG-UK

The number of samples in the COG-UK dataset collected between 16^th^ November 2020 and 10^th^ January 2021 from community areas, local to participating hospitals (i.e. shared adm2 designation), was tallied by week[^21^](https://www.zotero.org/google-docs/?KqD5Po).

Statistical analysis

Differences between patient groups in the prevalence of the Alpha variant among positive samples were evaluated using mixed effects logistic regression[^22^](https://www.zotero.org/google-docs/?rinGlH). CAI or HCAI, sex, age and sample week were included as predictive variables. Parameters for sample weeks were fitted separately for London sites compared with other sites grouped, and random intercept terms were included for each hospital and for weekly periods nested within hospitals. This analysis was also repeated including only the London sites.

Outbreak analyses were conducted using sequences with greater than 90% coverage across the SARS-CoV-2 genome (1043 sequences). Sequence diversity was measured by pairwise distance, defined as the number of single nucleotide polymorphisms (SNPs) differences between two sequences (excluding Ns), calculated in the R `ape` package[^23^](https://www.zotero.org/google-docs/?6cPr9V). The summary results were then grouped by lineage. To determine whether sequences were part of a nosocomial outbreak, we only focused on probable/definite HCAIs diagnosed ≥8 days post-admission. Cases occurring on the same wards (excluding known COVID-19 wards), with a pairwise distance of 0 (i.e. identical sequences) and within a time window of ⩽ 7 days were considered linked and part of the same outbreak. We also included, as independent outbreaks, all samples not linked to any other (i.e. one unlinked sample irrespective of time and location will count as an outbreak of size 1). As these patients all acquired the infection in hospital, they are likely to represent nosocomial transmission (for example from other patients or HCWs whose virus was not sequenced or did not achieve adequate coverage).

All analyses were conducted in R version 4.0.2, using tidyverse collection of packages and other statistical packages such as lme4[^22^](https://www.zotero.org/google-docs/?NwOMub), jtools[^24^](https://www.zotero.org/google-docs/?15CL6A) and rcompanion[^25^](https://www.zotero.org/google-docs/?iNBwoG). All plots were generated using ggplot2[^26^](https://www.zotero.org/google-docs/?KsAAdw).

**Results**

Study dataset

Between November 16^th^ 2020 and January 10^th^ 2021 SARS-CoV-2 RNA positive upper respiratory tract samples from 4184 subjects were successfully sequenced, including 2455 inpatients, 450 outpatients, 1166 HCWs and 113 (4.4 %) with unknown status. Of the inpatients, 1666 (64.9 %) were hospitalised with community-acquired infection, 215 (8.4 %) with indeterminate HCAI and 574 (22.4 %) with probable/definite HCAI, (Table 1). In total, 2058 samples were the Alpha variant, 4 samples were the Beta variant (lineage B.1.351) and 2122 were of lineages not designated variants of concern.The two most prominent lineages across the dataset were B.1.1.7 (the Alpha variant) and B.1.177. This was also true when restricting to HCAI samples alone (Supplementary Figure 1).

Data from laboratories not using Ct or equivalent thresholds confirmed that the proportions of the Alpha variant and non-Alpha variant viruses did not differ in samples with Ct values <=32 (Supplementary Figure 2, Chi-square test p=0.16).

Prevalence of the Alpha variant

The prevalence of the Alpha variant was highest in London and Hampshire (South of England), but substantially increased at all sites over the study period (Figure 1). On mixed effects logistic regression analysis of the Alpha variant, using 4165 samples with complete metadata, samples from HCWs (OR 0.78, 95 CI% 0.60 to 1.01), indeterminate HCAIs (OR 0.45, 95 CI% 0.30 to 0.70) or probable/definite HCAI (0.45, 0.34 to 0.59) were less likely to be identified as the Alpha variant compared to CAIs than non-Alpha variant. Suggesting that the proportion of hospital-acquired infections due to the Alpha variant was lower in any given week than the proportion among those presenting to hospital with community-acquired infection. However, changes in the frequency of the Alpha variant in CAIs correlated with those in HCAIs on a regional basis (Pearson’s correlation coefficient in London 0.90, 95% CI: 0.54-0.98, p-value<0.01, outside London 0.88, 95% CI 0.45-0.98, p-value<0.05) (Supplementary figure 3a). This relationship was confirmed also between HCAIs and community data from the general population (Pillar2, Supplementary figure 3b). Following the rapid growth of the Alpha variant within the community and hospitals, we observed a decrease of other lineages. In particular, B.1.177, which was the dominant strain in Europe before November 2020[^27,28^](https://www.zotero.org/google-docs/?HyC2B3), showed a correlation between CAIs and HCAIs (overall correlation 0.85) and an opposite trend to the Alpha variant with frequencies decreasing overtime (Supplementary figure 4).

Pairwise distance in HCAI

To help define outbreaks within hospitals, we used the sequence diversity within outbreaks involving patients with defined probable/definite HCAIs. We first compared the genetic distance among the Alpha variant sequences and separately among non-Alpha variant sequences of the same lineage. We found the mean pairwise distance (measured as number of SNPs difference) was lower between the Alpha variant samples than between samples from other lineages (mean=6.75 SNPs (95% CI 6.74-6.78) vs mean=8.01 SNPs (95% CI 7.95-8.07), Mann-Whitney U test p <0.05, Supplementary Figure 5). We next considered only viruses from patients who had very likely acquired their infection in hospital (i.e. probable/definite HCAIs). Excluding wards that were used for cohorting COVID-19 patients, the mean pairwise distance between sequences from patients on the same ward was higher for the Alpha variant acquired in hospital than for non-Alpha (mean=1.95 SNPs (95% CI 1.64-2.27) vs mean = 0.71 SNPS (95% CI 0.635-0.78), Mann-Whitney U test p <0.05). However, for both the Alpha variant and non-Alpha variants the pairwise distance between samples in the same ward was low.

Outbreaks

Given the low diversity observed within wards, and in agreement with previous studies[^14^](https://www.zotero.org/google-docs/?b5jLUu), a stringent definition was applied to define linked infections. Samples were considered linked, and part of the same outbreak, when the the sequences were completely identical and occurred on the same ward within a period of 7 days. Outbreaks of size one, corresponding to samples not linked to any other sample, were allowed. The 7 day threshold is consistent with evidence that most people become symptomatic 7 days after exposure [^29,30^](https://www.zotero.org/google-docs/?SXn5Ci). This choice was also inline with previous transmission studies[^16^](https://www.zotero.org/google-docs/?vBPWef). The impact of allowing for multi-ward outbreaks and varying the time period and the pairwise SNP differences defining an outbreak was tested in a sensitivity analysis.

Ward data was available for a total of 497 probable/definite HCAI patients. A total of 83 outbreaks were identified (by the above definition) caused by any lineage across all hospitals, 19 of which were caused by the Alpha variant. Outbreaks caused by the Alpha variant in hospitals increased with time, associated with the changing prevalence of the Alpha variant within the community (Figure 2). In contrast outbreaks due to other lineages decreased in line with reduced circulation of those lineages in the community. Whilst this trend is observed both within and outside London, the dominance of the Alpha variant outbreaks occurs earlier within London, reflecting the earlier rise in the community.

The sizes of outbreak clusters within hospitals caused by the Alpha variant and by other lineages were compared. The total number of probable/definite HCAI patients in a single outbreak ranged from 1 to 11. There was no significant difference in the number of patients involved in outbreaks caused by the Alpha variant compared to outbreaks caused by other lineages (global Kruskal-Wallis p-value=0.27, pairwise comparisons non-significant, Figure 3). The mean size for the Alpha variant outbreaks was 2.22 in London (95% CI 1.22-3.22) and 3.30 in other locations (95% CI 1.39-5.21). Outbreaks of non-B.1.1.7 lineages had a mean size of 3.72 and 2.78 in London and outside respectively (95% CI 2.32-5.13 in London and 95% CI 2.08-3.49 outside). These conclusions were unchanged, by the sensitivity analyses (Supplementary Figure 6).

**Discussion**

Nosocomial transmission continues to present a major challenge to the control of SARS-CoV-2 infection. Overall SARS-CoV-2 acquired in hospitals is estimated to have accounted for up to 20% COVID-19 inpatient cases during the first wave[^31^](https://www.zotero.org/google-docs/?QBBgHj). Recent data from Scotland suggest that up to 36% of severe COVID-19 is associated with recent exposure in hospital (from 1 March 2020 to 28 January 2021)[^32^](https://www.zotero.org/google-docs/?IguX31). This is in line with the proportions identified in our data, with 22.4% of inpatients having probable/definite HCAI and 8.4% having indeterminate HCAI across all sites. The emergence of new variants with evidence of greater transmissibility in the community presents a potentially increased threat of nosocomial transmission leading to calls for better protection for staff and patients[^15^](https://www.zotero.org/google-docs/?NkaIRN).

Using detailed metadata on community and healthcare-acquired infections from 2455 inpatients in 9 hospitals across the UK linked to genomic data sequenced during the winter of 20/21 as part of COG-UK HOCI study, logistic regression analysis showed that having a healthcare-acquired infection was predictive of non-Alpha variants. This implies that the Alpha variant was not spreading faster within hospitals than in the community (Table 2). This finding was despite a rise in numbers of COVID-19 cases among both inpatients and the community, with an increasing proportion caused by the Alpha variant (Figure 1). As has been previously reported, the total numbers of HCAIs were closely correlated with the rising numbers of cases in the community and the increase in HCAI infections caused by the Alpha variant also correlated with increasing prevalence of the Alpha variant overall[^29^](https://www.zotero.org/google-docs/?ONQM7d).

We made use of the genomic data and detailed information on hospital acquired infections to better identify and quantify linked hospital infections. The definition of an outbreak was considered carefully. Previous outbreak data suggest that the mutation rate of SARS-CoV-2 is low, with an average of less than one fixed mutation occurring for each transmission[^33^](https://www.zotero.org/google-docs/?Dhv3pC). Nonetheless, up to 2 single nucleotide differences have been described in viruses that are known to be part of a single nosocomial outbreak[^34^](https://www.zotero.org/google-docs/?anYo9U). In our data, we noted very little genetic diversity across the Alpha variant (Supplementary figure 5), reflecting the rapid expansion and selective sweep that occurred as the variant rapidly spread. We therefore chose a stringent definition of linked infections, requiring identical sequences and included only patients with a high likelihood of having acquired their infection in hospital (i.e. probable or definite hospital onset SARS-CoV-2 infection). We also restricted putatively linked cases to those on the same ward and within a time window of 7 days to further increase the specificity of outbreak definition. Within these constraints, the genomic data failed to identify a difference between the size of outbreaks occurring on wards between the Alpha variant and previously circulating lineages.

However, the outbreak definition implemented in our primary analysis is rather stringent. First, as we lack complete records of patients movement, we potentially exclude linked cases in different wards, for example patients who were infected by the same health-care worker or patients who moved before/after diagnosis. Second, our choice of a 7 days window is rather conservative, considering that estimates of the incubation period vary with some outbreak studies opting for a larger period of 14 days[^29^](https://www.zotero.org/google-docs/?ou6sgJ). Third, using only identical sequences we could bias against lineages with smaller diversity. To assess the impact of our parameters’ choice and the robustness of our results, we carried out a sensitivity analysis varying our parameters to link cases. Allowing for multi-ward outbreaks, increasing the numbers of SNP differences to two and varying the time interval for defining linked cases (0, 7 and 14 days) failed to change the findings.

There are a number of limitations to our work. First, we were not able to sequence all positive cases. Five of nine centres only sequenced samples with PCR cycle thresholds of 32 and below i.e. higher viral loads. Notably though, sequencing of 694 cases, from three labs not using Ct thresholds with available Ct data, did not find any difference in the distribution of genotypes in samples with Ct values below and above 32 (supplementary figure 1). A second limitation of our work is that towards the end of the study all three trusts outside London were using a sequence reporting tool (SRT), as part of the HOCI study[^33^](https://www.zotero.org/google-docs/?k6zPPP), rather than phylogenetic analysis alone to help determine whether cases were part of linked outbreaks. It is not known whether the SRT may have limited the extent of outbreaks as data processing and analysis for the HOCI study is still ongoing. Finally, this study was not designed to account for use of personal protective equipment (PPE), aerosol generating procedures (AGP) or ventilation which may also impact transmission.

In summary notwithstanding its greater transmissibility in the community, we find no evidence to support the Alpha variant as having caused more nosocomial transmission than previous variants. This suggests that the stringent infection prevention measures already in place in UK hospitals are similarly effective at containing the spread of SARS-CoV-2 in a healthcare setting irrespective of its transmissibility. This finding implies that ongoing nosocomial spread of SARS-CoV-2 is likely to be influenced by factors such as fixed estate, e.g. building infrastructure, beds in bays, shared facilities and ventilation, which are not readily mitigated by the existing infection prevention and control (IPC) measures. However, there is some reassurance that currently implemented IPC measures are likely to be as effective against more transmissible variants.

**References**

[1 Volz E, Mishra S, Chand M, *et al.* Transmission of SARS-CoV-2 Lineage B.1.1.7 in England: Insights from linking epidemiological and genetic data. *medRxiv* 2021; : 2020.12.30.20249034.](https://www.zotero.org/google-docs/?ynL1xC)

[2 Davies NG, Abbott S, Barnard RC, *et al.* Estimated transmissibility and severity of novel SARS-CoV-2 Variant of Concern 202012/01 in England. *medRxiv* 2021; : 2020.12.24.20248822.](https://www.zotero.org/google-docs/?ynL1xC)

[3 PANGO lineages. https://cov-lineages.org/global_report.html (accessed Feb 9, 2021).](https://www.zotero.org/google-docs/?ynL1xC)

[4 Lineage-specific growth of SARS-CoV-2 B.1.1.7 during the English national lockdown - SARS-CoV-2 coronavirus / nCoV-2019 Genomic Epidemiology. Virological. 2020; published online Dec 30. https://virological.org/t/lineage-specific-growth-of-sars-cov-2-b-1-1-7-during-the-english-national-lockdown/575 (accessed Feb 8, 2021).](https://www.zotero.org/google-docs/?ynL1xC)

[5 Confirmed cases of COVID-19 variants identified in UK. GOV.UK. https://www.gov.uk/government/news/confirmed-cases-of-covid-19-variants-identified-in-uk (accessed June 29, 2021).](https://www.zotero.org/google-docs/?ynL1xC)

[6 SPI-M-O: Consensus statement on COVID-19, 3 June 2021. GOV.UK. https://www.gov.uk/government/publications/spi-m-o-consensus-statement-on-covid-19-3-june-2021 (accessed June 29, 2021).](https://www.zotero.org/google-docs/?ynL1xC)

[7 Kemp SA, Collier DA, Datir RP, *et al.* SARS-CoV-2 evolution during treatment of chronic infection. *Nature* 2021; : 1–10.](https://www.zotero.org/google-docs/?ynL1xC)

[8 Wise J. Covid-19: New coronavirus variant is identified in UK. *BMJ* 2020; **371**: m4857.](https://www.zotero.org/google-docs/?ynL1xC)

[9 Starr TN, Greaney AJ, Dingens AS, Bloom JD. Complete map of SARS-CoV-2 RBD mutations that escape the monoclonal antibody LY-CoV555 and its cocktail with LY-CoV016. *Cell Rep Med* 2021; **2**: 100255.](https://www.zotero.org/google-docs/?ynL1xC)

[10 Zhang W, Davis BD, Chen SS, Sincuir Martinez JM, Plummer JT, Vail E. Emergence of a Novel SARS-CoV-2 Variant in Southern California. *JAMA* 2021; **325**: 1324.](https://www.zotero.org/google-docs/?ynL1xC)

[11 Rickman HM, Rampling T, Shaw K, *et al.* Nosocomial Transmission of Coronavirus Disease 2019: A Retrospective Study of 66 Hospital-acquired Cases in a London Teaching Hospital. *Clin Infect Dis* 2021; **72**: 690–3.](https://www.zotero.org/google-docs/?ynL1xC)

[12 Wang D, Hu B, Hu C, *et al.* Clinical Characteristics of 138 Hospitalized Patients With 2019 Novel Coronavirus–Infected Pneumonia in Wuhan, China. *JAMA* 2020; **323**: 1061–9.](https://www.zotero.org/google-docs/?ynL1xC)

[13 Bhattacharya A, Collin SM, Stimson J, *et al.* Healthcare-associated COVID-19 in England: a national data linkage study. Infectious Diseases (except HIV/AIDS), 2021 DOI:10.1101/2021.02.16.21251625.](https://www.zotero.org/google-docs/?ynL1xC)

[14 Meredith LW, Hamilton WL, Warne B, *et al.* Rapid implementation of SARS-CoV-2 sequencing to investigate cases of health-care associated COVID-19: a prospective genomic surveillance study. *Lancet Infect Dis* 2020; **20**: 1263–71.](https://www.zotero.org/google-docs/?ynL1xC)

[15 Oliver D. David Oliver: Could we do better on hospital acquired covid-19 in a future wave? *BMJ* 2021; **372**: n70.](https://www.zotero.org/google-docs/?ynL1xC)

[16 Duverger C, Souyri V, Monteil C, *et al.* Controlling healthcare-associated transmission of SARS-CoV-2 variant of concern 202012/01 in a large hospital network. *J Hosp Infect* 2021; **0**. DOI:10.1016/j.jhin.2021.04.031.](https://www.zotero.org/google-docs/?ynL1xC)

[17 Stirrup OT, Boshier FAT, Venturini C, *et al.* SARS-CoV-2 lineage B.1.1.7 is associated with greater disease severity among hospitalised women but not men. *medRxiv* 2021; : 2021.06.24.21259107.](https://www.zotero.org/google-docs/?ynL1xC)

[18 COVID-19: epidemiological definitions of outbreaks and clusters. GOV.UK. https://www.gov.uk/government/publications/covid-19-epidemiological-definitions-of-outbreaks-and-clusters (accessed Feb 9, 2021).](https://www.zotero.org/google-docs/?ynL1xC)

[19 Protocols | COVID-19 Genomics UK Consortium. COVID-19 Genomics UK Consort. UK-Wide Genomic Seq. 2021; published online Jan 12. https://www.cogconsortium.uk/tools-analysis/public-data-analysis/ (accessed July 6, 2021).](https://www.zotero.org/google-docs/?ynL1xC)

[20 cov-lineages/pangolin. CoV-lineages, 2021 https://github.com/cov-lineages/pangolin (accessed Feb 9, 2021).](https://www.zotero.org/google-docs/?ynL1xC)

[21 Home - COG-UK Consortium. https://www.cogconsortium.uk/ (accessed Feb 10, 2021).](https://www.zotero.org/google-docs/?ynL1xC)

[22 Bates D, Mächler M, Bolker B, Walker S. Fitting Linear Mixed-Effects Models Using lme4. *J Stat Softw* 2015; **67**: 1–48.](https://www.zotero.org/google-docs/?ynL1xC)

[23 Paradis E, Schliep K. ape 5.0: an environment for modern phylogenetics and evolutionary analyses in R. *Bioinformatics* 2019; **35**: 526–8.](https://www.zotero.org/google-docs/?ynL1xC)

[24 Analysis and Presentation of Social Scientific Data. https://jtools.jacob-long.com/ (accessed June 16, 2021).](https://www.zotero.org/google-docs/?ynL1xC)

[25 Mangiafico S. Functions to Support Extension Education Program Evaluation [R package rcompanion version 2.4.1]. 2021; published online May 18. https://CRAN.R-project.org/package=rcompanion (accessed June 16, 2021).](https://www.zotero.org/google-docs/?ynL1xC)

[26 Wickham H, Averick M, Bryan J, *et al.* Welcome to the Tidyverse. *J Open Source Softw* 2019; **4**: 1686.](https://www.zotero.org/google-docs/?ynL1xC)

[27 Di Giallonardo F, Puglia I, Curini V, *et al.* Emergence and Spread of SARS-CoV-2 Lineages B.1.1.7 and P.1 in Italy. *Viruses* 2021; **13**: 794.](https://www.zotero.org/google-docs/?ynL1xC)

[28 Recombinant SARS-CoV-2 genomes involving lineage B.1.1.7 in the UK - SARS-CoV-2 coronavirus / SARS-CoV-2 Molecular Evolution. Virological. 2021; published online March 17. https://virological.org/t/recombinant-sars-cov-2-genomes-involving-lineage-b-1-1-7-in-the-uk/658 (accessed May 11, 2021).](https://www.zotero.org/google-docs/?ynL1xC)

[29 Snell LB, Fisher CL, Taj U, *et al.* Combined epidemiological and genomic analysis of nosocomial SARS-CoV-2 transmission identifies community social distancing as the dominant intervention reducing outbreaks. *medRxiv* 2020; : 2020.11.17.20232827.](https://www.zotero.org/google-docs/?ynL1xC)

[30 Wiersinga WJ, Rhodes A, Cheng AC, Peacock SJ, Prescott HC. Pathophysiology, Transmission, Diagnosis, and Treatment of Coronavirus Disease 2019 (COVID-19): A Review. *JAMA* 2020; **324**: 782–93.](https://www.zotero.org/google-docs/?ynL1xC)

[31 The impact of testing and infection prevention and control strategies on within-hospital transmission dynamics of COVID-19 in English hospitals | Philosophical Transactions of the Royal Society B: Biological Sciences. https://royalsocietypublishing.org/doi/full/10.1098/rstb.2020.0268 (accessed June 25, 2021).](https://www.zotero.org/google-docs/?ynL1xC)

[32 Relation of severe COVID-19 in Scotland to transmission-related factors and risk conditions eligible for shielding support: REACT-SCOT case-control study | medRxiv. https://www.medrxiv.org/content/10.1101/2021.03.02.21252734v1 (accessed June 25, 2021).](https://www.zotero.org/google-docs/?ynL1xC)

[33 Stirrup O, Hughes J, Parker M, *et al.* Rapid feedback on hospital onset SARS-CoV-2 infections combining epidemiological and sequencing data. *eLife* 2021; **10**: e65828.](https://www.zotero.org/google-docs/?ynL1xC)

[34 Rockett RJ, Arnott A, Lam C, *et al.* Revealing COVID-19 transmission in Australia by SARS-CoV-2 genome sequencing and agent-based modeling. *Nat Med* 2020; **26**: 1398–404.](https://www.zotero.org/google-docs/?ynL1xC)

**Table 1** Proportion of SARS-CoV-2 due to the Alpha variant for all sequenced samples

|  | **Alpha variant (n=2058)** | **Non-Alpha variant (n=2126)** | **Total (n=4184)** |
| --- | --- | --- | --- |
| **Age [mean (sd)]** |  |  |  |
|  | 53.4 (21.8) | 58 (22.6) | 55.7 (22.3) |
| missing | 0 | 1 | 1 |
| **Sex** |  |  |  |
| Female | 1109 (48.6) | 1175 (51.4) | 2,284 (100.0) |
| Male | 938 (49.8) | 944 (50.2) | 1,882 (100.0) |
| missing | 11 | 7 | 18 |
| **Week starting:** |  |  |  |
| 16/11/2020 | 22 (8.5) | 238 (91.5) | 260 (100.0) |
| 23/11/2020 | 50 (15.0) | 284 (85.0) | 334 (100.0) |
| 30/11/2020 | 83 (20.4) | 324 (79.6) | 407 (100.0) |
| 07/12/2020 | 128 (30.0) | 299 (70.0) | 427 (100.0) |
| 14/12/2020 | 312 (45.7) | 370 (54.3) | 682 (100.0) |
| 21/12/2020 | 411 (57.2) | 307 (42.8) | 718 (100.0) |
| 28/12/2020 | 648 (75.2) | 214 (24.8) | 862 (100.0) |
| 04/01/2021 | 404 (81.8) | 90 (18.2) | 494 (100.0) |
| **Patient Class** |  |  |  |
| Outpatients | 250 (55.6) | 200 (44.4) | 450 (100.0) |
| Any HCW | 559 (47.9) | 607 (52.1) | 1,166 (100.0) |
| Inpatients | 1182 (48.1) | 1273 (51.9) | 2,455 (100.0) |
| CAI* | 926 (55.6) | 740 (44.4) | 1,666 (100.0) |
| Indeterminate HCAI† | 56 (26.0) | 159 (74.0) | 215 (100.0) |
| Probable/definite HCAI‡ | 200 (34.8) | 374 (65.2) | 574 (100.0) |
| Unknown category | 67 (59.3) | 46 (40.7) | 113 (100.0) |
| **Region** |  |  |  |
| Glasgow | 91 (31.6) | 197 (68.4) | 288 (100.0) |
| Hampshire | 288 (66.2) | 147 (33.8) | 435 (100.0) |
| London | 1480 (65.6) | 775 (34.4) | 2,255 (100.0) |
| South Yorkshire | 199 (16.5) | 1007 (83.5) | 1,206 (100.0) |

*Diagnosed at or ≤2 days from admission. †Diagnosed 3-7 days from admission. ‡Diagnosed ≥8 days from admission. CAI, community-acquired infection; HCAI, healthcare-associated infection; HCW, healthcare worker.**Table 2** Multivariable mixed effects logistic regression for prediction of being infected with the Alpha variant among positive samples sequenced by hospital labs.

|  | **All samples** | |  | **London sites only** | |
| --- | --- | --- | --- | --- | --- |
|  | **OR (95% CI)** | **p-value** |  | **OR (95% CI)** | **p-value** |
| **Age** |  | 0.03 |  |  | 0.09 |
|  | 0.99  (0.99 to 1.00) |  |  | 1.00  (0.99 to 1.00) |  |
| **Sex** |  | 0.51 |  |  | 0.43 |
| Female | *Reference* |  |  | *Reference* |  |
| Male | 0.95  (0.80 to 1.12) |  |  | 0.92  (0.74 to 1.14) |  |
| **Patient class** |  | <0.001 |  |  | <0.001 |
| Inpatient (CAI)† | *Reference* |  |  | *Reference* |  |
| A&E attendee | 1.35  (0.87 to 2.09) |  |  | 1.25 (0.76 to 2.05) |  |
| Outpatient | 0.86  (0.58 to 1.26) |  |  | 0.78 (0.47 to 1.32) |  |
| Any HCW | 0.78  (0.60 to 1.01) |  |  | 0.67 (0.48 to 0.93) |  |
| Indeterminate HCAI‡ | 0.45  (0.30 to 0.70) |  |  | 0.33 (0.19 to 0.58) |  |
| Probable/definite HCAI⁋ | 0.45  (0.34 to 0.59) |  |  | 0.29 (0.20 to 0.41) |  |
| Unknown | 2.46  (1.41 to 4.30) |  |  | 3.19 (1.28 to 7.92) |  |
| **Week starting:** | **Mean Prop. London#** | **Mean Prop. Elsewhere#** |  | **Mean Prop.#** |  |
| 16/11/2020 | 0.14  (0.09 to 0.21) | 0.02  (0 to 0.07) |  | 0.08  (0.06 to 0.13) |  |
| 23/11/2020 | 0.23  (0.17 to 0.30) | 0.03  (0.01 to 0.08) |  | 0.15  (0.11 to 0.19) |  |
| 30/11/2020 | 0.36  (0.30 to 0.44) | 0.05  (0.03 to 0.09) |  | 0.20  (0.17 to 0.25) |  |
| 07/12/2020 | 0.50  (0.43 to 0.57) | 0.10  (0.07 to 0.15) |  | 0.30  (0.26 to 0.35) |  |
| 14/12/2020 | 0.76  (0.67 to 0.81) | 0.18  (0.14 to 0.22) |  | 0.46  (0.42 to 0.50) |  |
| 21/12/2020 | 0.77  (0.72 to 0.81) | 0.30  (0.25 to 0.36) |  | 0.57  (0.53 to 0.60) |  |
| 28/12/2020 | 0.86  (0.83 to 0.89) | 0.60  (0.55 to 0.65) |  | 0.75  (0.72 to 0.78) |  |
| 04/01/2021 | 0.88  (0.84 to 0.92) | 0.74  (0.67 to 0.79) |  | 0.82  (0.78 to 0.84) |  |

†Diagnosed at or ≤2 days from admission. ‡Diagnosed 3-7 days from admission. ⁋Diagnosed ≥8 days from admission. **#**Estimate of proportion infected with the Alpha variant from model for a 55-year-old male inpatient admitted with COVID-19. CAI, community-acquired infection; HCAI, healthcare-associated infection; HCW, healthcare worker; OR, odds ratio.

**Figure 1** Prevalence over time of the Alpha variant in hospitalized patients, healthcare workers (HCWs) and community samples (Pillar 2 data as described in methods) from different geographical regions in the UK. Hospitalized patients are displayed according to community-acquired infection (CAI) (diagnosed at or ≤2 days from admission) or healthcare-associated infection (HCAI) (diagnosed ≥3 days from admission).


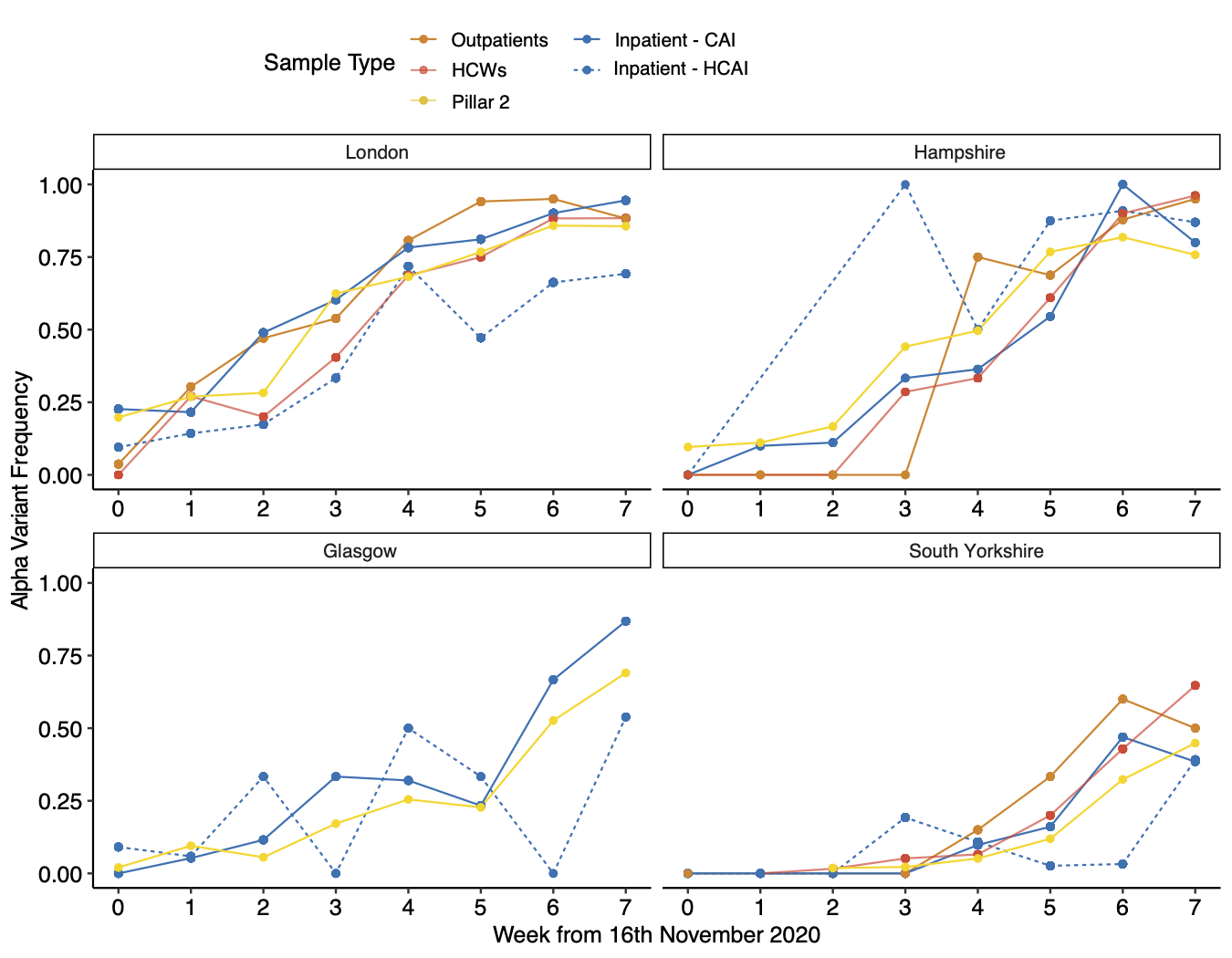


**Figure 2** Barplot showing number of HOCI patients involved in outbreaks by week and location, coloured by variant (Alpha vs non-Alpha). Line-chart represents the number of CAI (community-acquired infections, including inpatients, outpatient, A&E patients and healthcare workers) overtime coloured by variant (Alpha variant presence/absence).

*
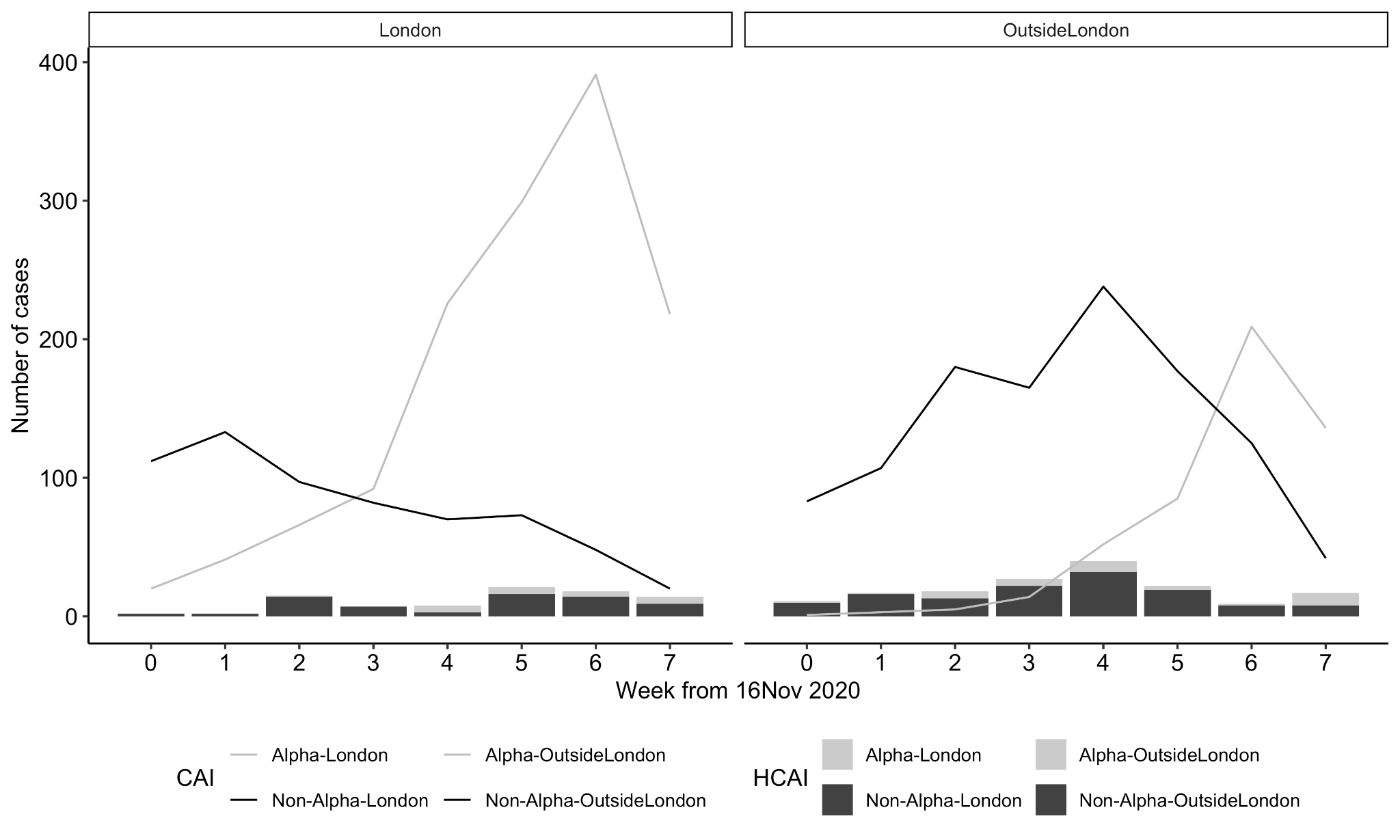
*

**Figure 3** Violin plot showing the size of outbreaks in hospital-onset COVID-19 infection patients for four categories: outbreaks caused by the Alpha variant in London and other locations and outbreaks caused by other lineages in London and outside London. Colour represents lineages: in lighter grey the Alpha variant and in black non-Alpha variant. Non-parametric global Kruskal-Wallis p-value=0.27, pairwise comparisons (Mann-Whitney) non-significant. The number below each violin shows the number of clusters/outbreaks for that category.

**
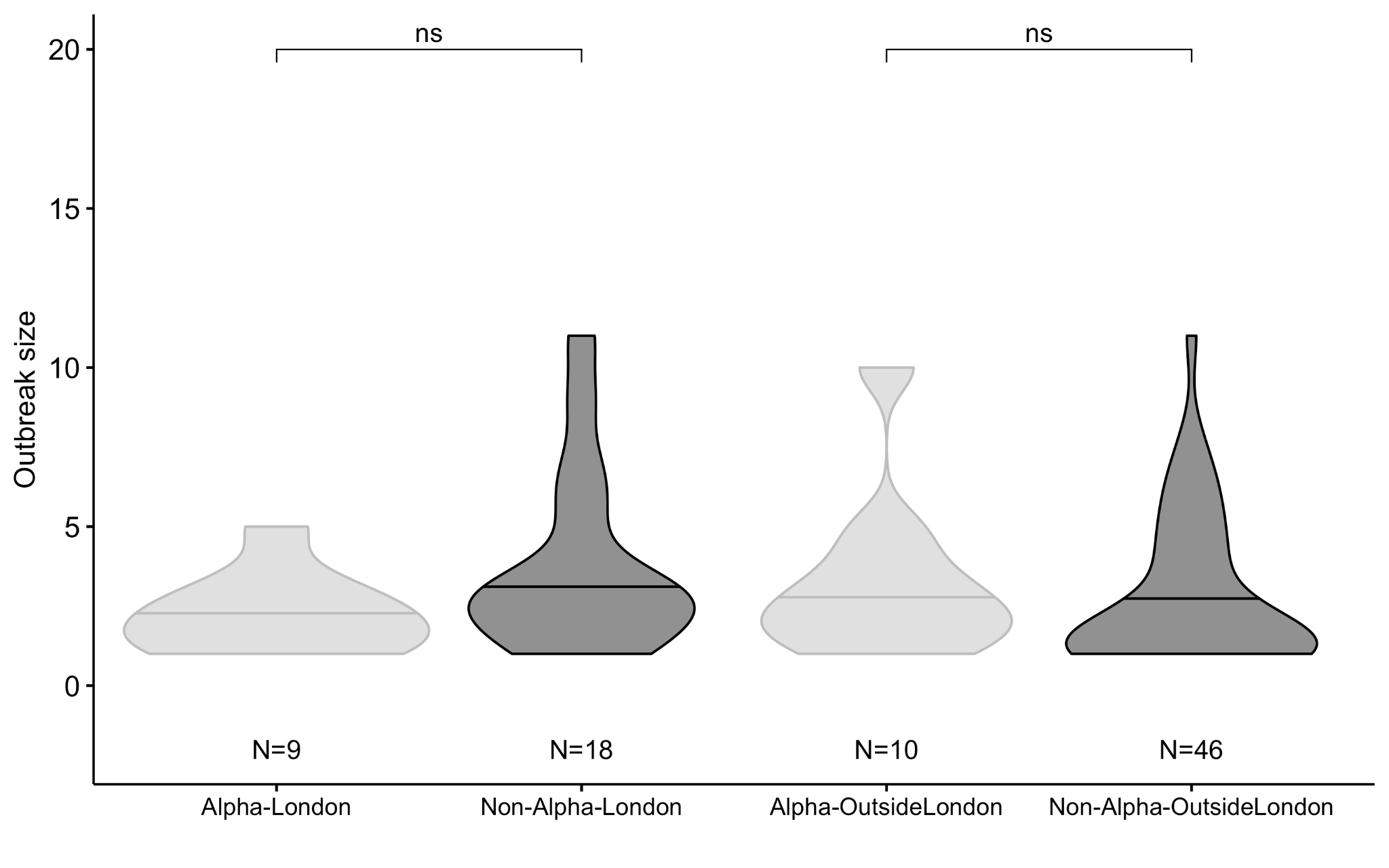
**

**Acknowledgments**

This report was produced by members of the **COG-UK HOCI Variant substudy consortium**. **COG-UK HOCI** is part of **COG-UK**. COG-UK is supported by funding from the Medical Research Council (MRC) part of UK Research & Innovation (UKRI), the National Institute of Health Research (NIHR) and Genome Research Limited, operating as the Wellcome Sanger Institute.

**SUPPLEMENTARY MATERIAL**

**Supplementary Figure 1.** Proportion of lineages with >5% frequency in all samples and inpatients with HCAI. The two most frequent lineages are B.1.1.7 (the Alpha variant) and B.1.177 in both populations. The number by each segment represents the sample size.

**
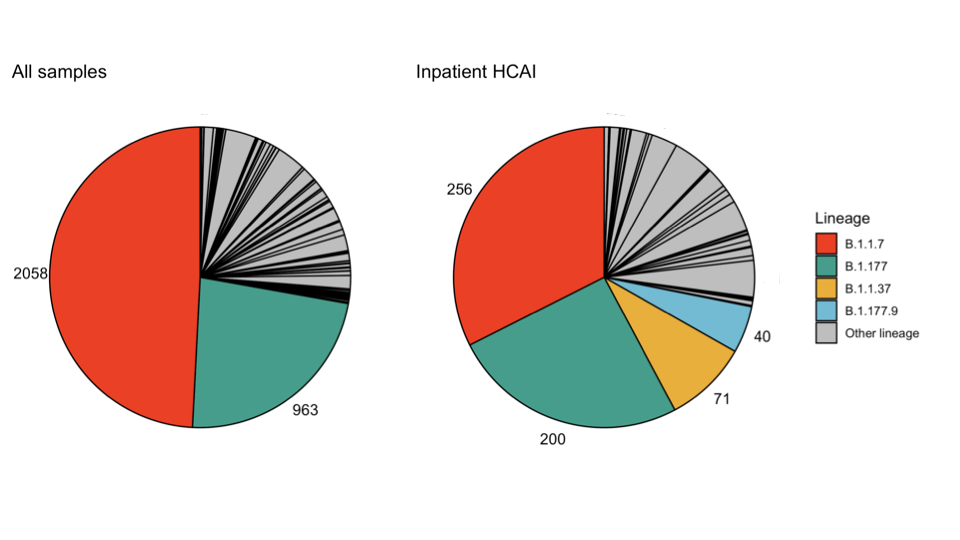
**

**Supplementary Figure 2** Proportions of the Alpha variant and non-Alpha variants sequencing successfully at Ct values above and below 32 for 3 trusts not using a Ct threshold. No significant difference was found in the proportion of lineages detected in each category. Number of samples in each category are inlaid in each bar.


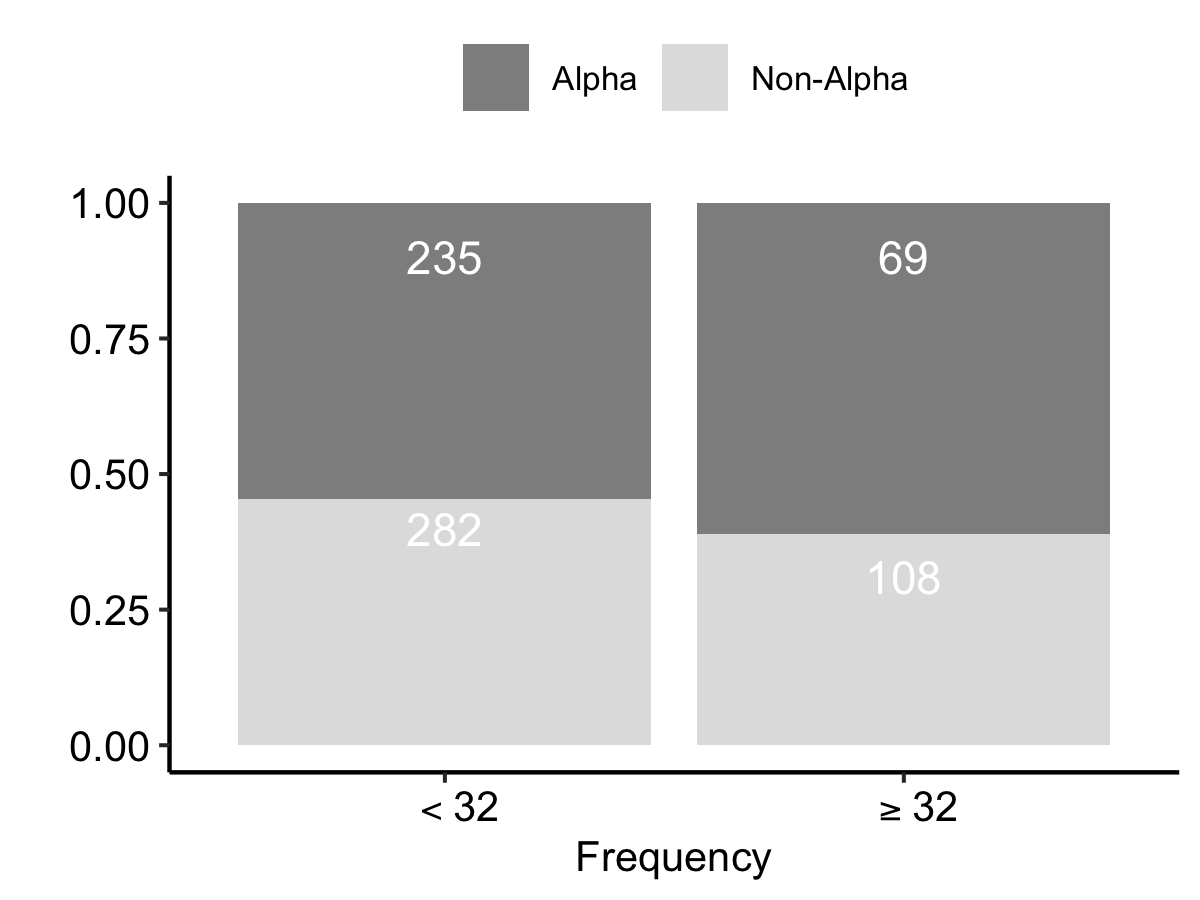


**Supplementary figure 3. Correlation of Alpha variant proportions in HCAI inpatients (only probable/definite HCAI) and (a) CAIand (b) in Pillar2 by week and location.** Each dot represents a week counting from 16th November 2020. In (a) the overall correlation is 0.89 (95% CI: 0.71-0.96), p-value=3.712e-06. In London it is 0.90 (95% CI: 0.54-0.98), p-value=0.002 and outside London is 0.88 (95% CI: 0.45-0.98), p-value=0.004. In (b) the overall correlation is 0.87 (95% CI: 0.67-0.96) p-value=9.461e-06. In London it is 0.91 (95% CI: 0.58-0.98), p-value=0.001 and outside London is 0.75 (95% CI: 0.11-0.95), p-value=0.031.

**a)
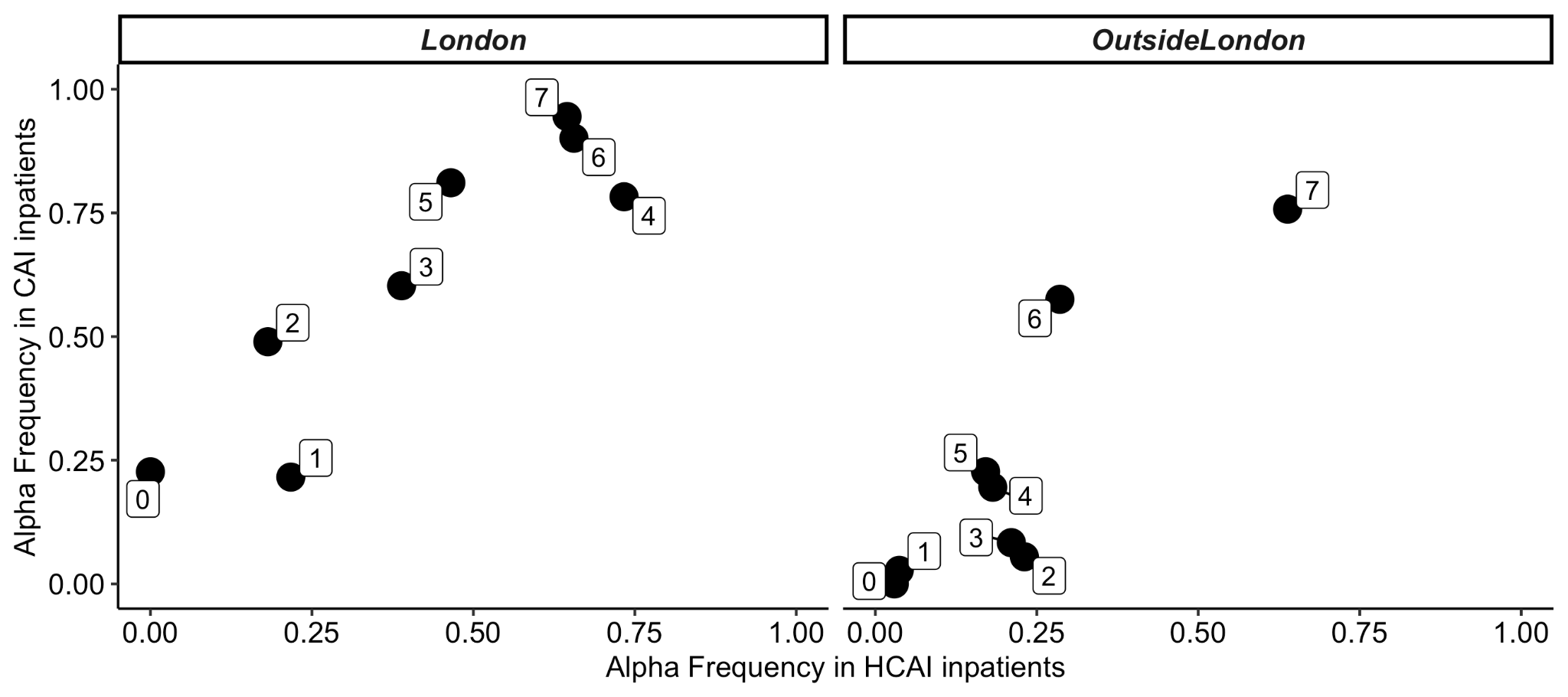
**

**b)
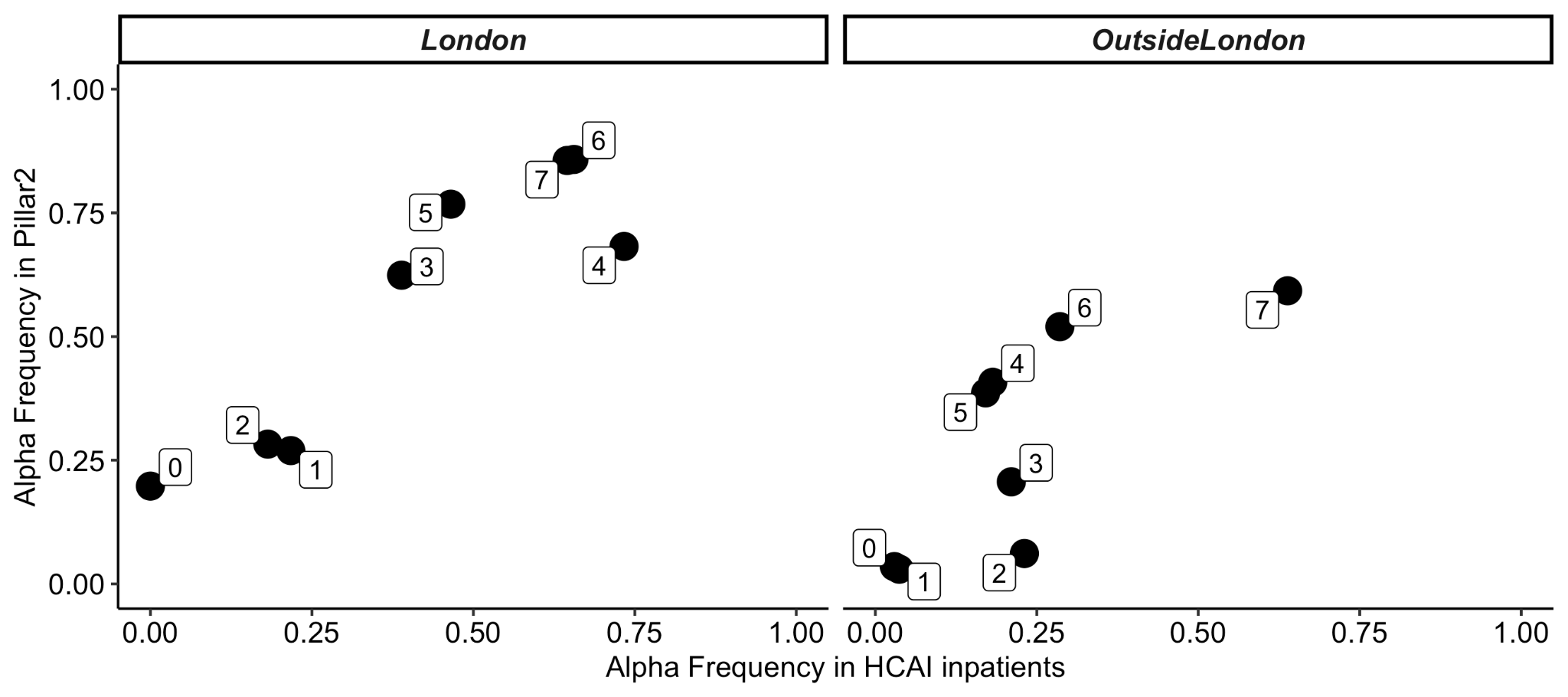
**

**Supplementary figure 4. Correlation B.1.177 (non-Alpha variant) proportions in healthcare-acquired infection (HCAI) inpatients (only probable/definite HCAI) and community-acquired infection (CAI) by week and location.** The overall correlation is 0.85 (95% CI: 0.62-0.95), p-value=2.791e-05. In London it is 0.84 (95% CI: 0.32-0.97), p-value=0.01 and outside London is 0.7 (95% CI: -0.005-0.94), p-value=0.052.

**
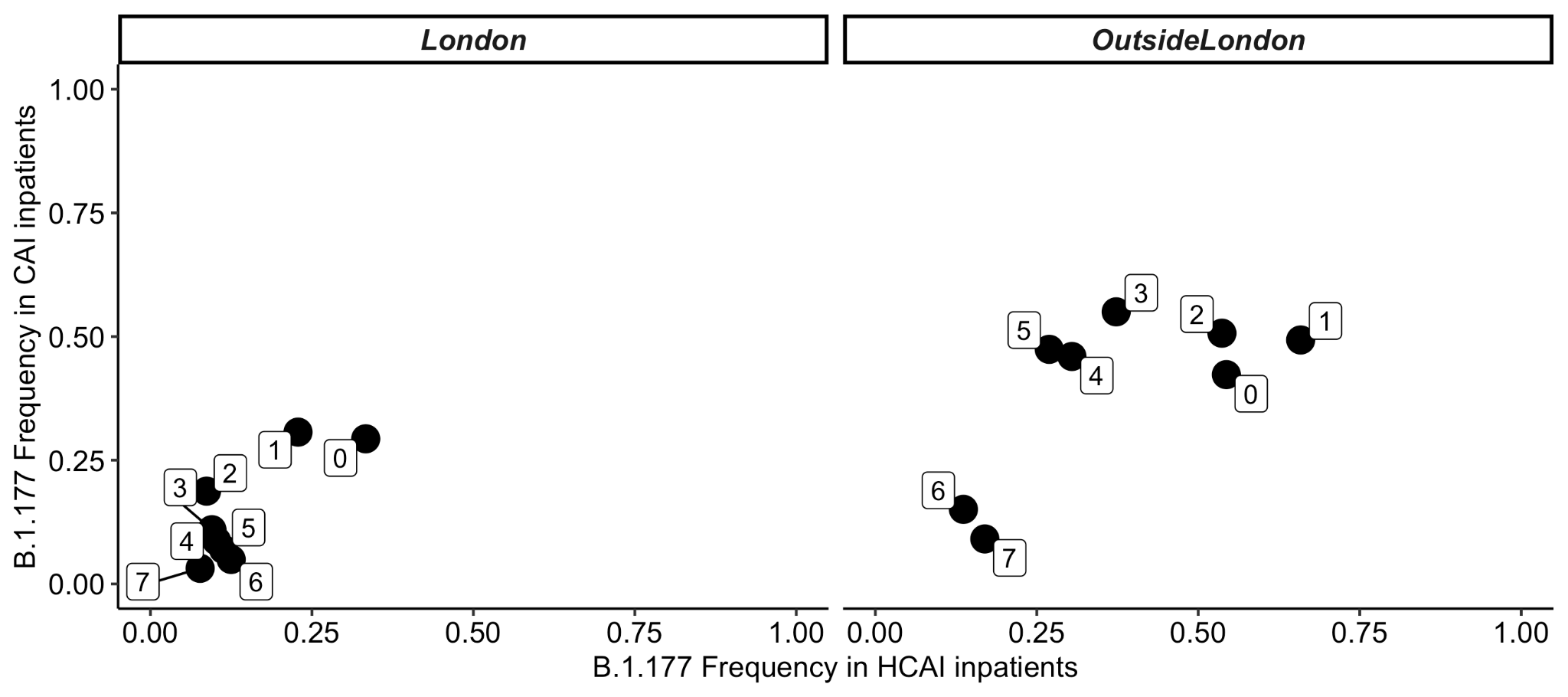
**

**Supplementary Figure 5** Violin plot of pairwise distance of probable/definite HCAI samples with 90% coverage from the Alpha variant and non-Alpha variant a) across all samples b) within samples in the same trust and ward. The mean pairwise distance of HCAI samples was lower for the Alpha variant than non-Alpha variant (mean=6.75 SNPs (95% CI 6.74-6.78) vs mean=8.01 SNPs (95% CI 7.95-8.07), Mann-Whitney U test p <0.05). The mean pairwise distance between probable/definite HCAI samples taken from the same trust and ward was higher for the Alpha variant than non-Alpha variant (mean=1.95 SNPs (95% CI 1.64-2.27) vs mean = 0.71 SNPs (95% CI 0.64-0.78), Mann-Whitney U test p <0.05). The number below the violin plots indicates the corresponding number of pairwise comparisons.

**
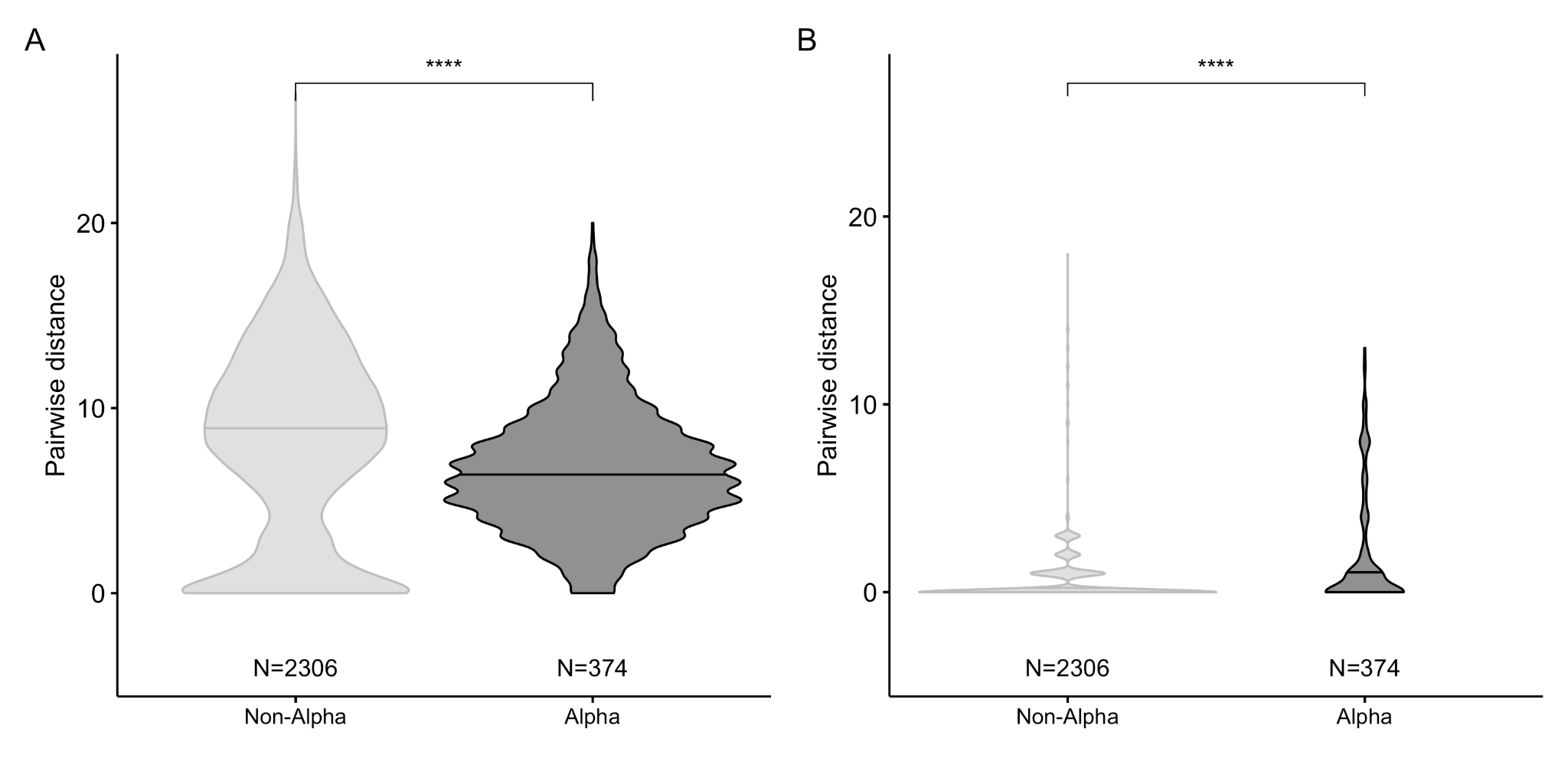
**

**Supplementary Figure 6: Sensitivity analysis comparing different outbreak/cluster definitions.** The violin plot shows the size of outbreaks in hospital-onset COVID-19 infection patients for four categories: the Alpha variant in hospitals in London and outside London, non-Alpha variant in London and outside London. Different outbreak’s definitions are presented: A) same lineage, no time threshold (A1), 7-days threshold, (A2) 14 days threshold (A3); B) SNPs difference = 0 (identical sequence), no time threshold (B1), 7-days threshold (same as figure 3), (B2) 14 days threshold (B3); C) SNPs difference <=2, no time threshold (C1), 7-days threshold (same as figure 3), (C2) 14 days threshold (C3); D) SNPs difference = 0 multi-ward within hospital , no time threshold (D1), 7-days threshold (D2), 14 days threshold (D3).


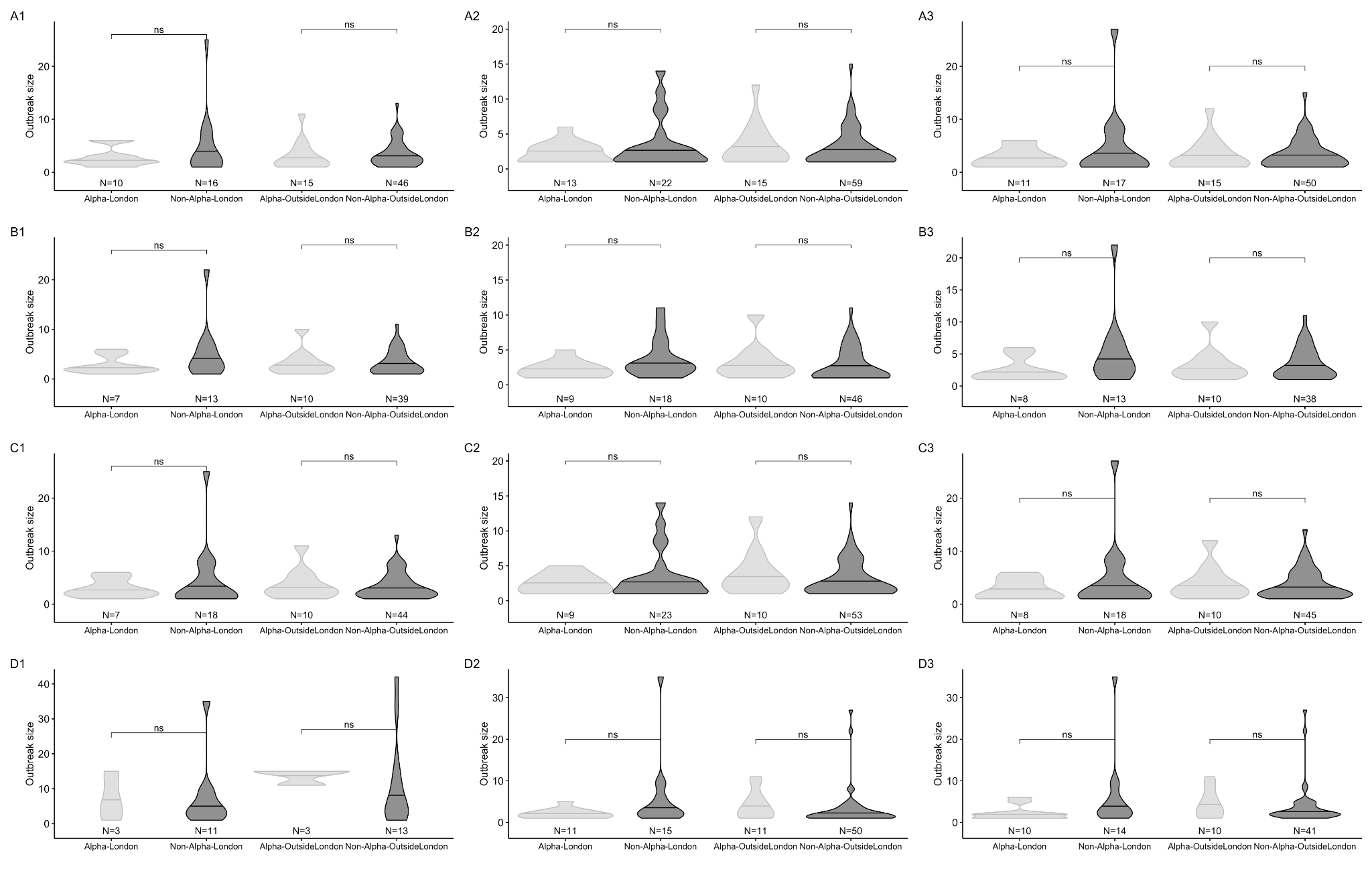

Supplement: Supplementary file 2 [file mmc2.docx]
